# EFCD Expert Non-Consensus on Material and Technique Use for Direct Class II Composites With Guidance on Restoring Complex Cavities

**DOI:** 10.3290/j.jad.c_2836

**Published:** 2026-10-02

**Authors:** Oliver Bailey, Frank Pfefferkorn, Thomas Attin, Roland Frankenberger, Bas Loomans, Anne-Katrin Lührs, Nicola Scotti, Diana Wolff, Reinhard Hickel, Niek Opdam

**Affiliations:** a Oliver Bailey Clinical Lecturer, School of Dental Sciences, Newcastle University, Newcastle upon Tyne, UK. Provided the initial draft of the manuscript, plus contributed to the idea, methods, analysis and critically evaluated the manuscript.; b Frank Pfefferkorn Senior Clinical Research Manager, Dentsply Sirona, Clinical Affairs & Workflows, Dentsply DeTrey GmbH, DeTrey-Str. 1, 78467 Konstanz, Germany. Contributed to the idea, methods, analysis and critically evaluated the manuscript.; c Thomas Attin Chairman, Clinic of Conservative and Preventive Dentistry, Center for Dental Medicine, University Zurich, Plattenstr. 11, CH-8032 Zurich, Switzerland. Contributed to the idea, methods, analysis and critically evaluated the manuscript.; d Roland Frankenberger Professor and Chair, Department of Operative Dentistry, Endodontics, and Pediatric Dentistry, University Dental Medicine, University of Marburg and University Hospital Giessen and Marburg, Campus Marburg, Georg Voigt St 3, D-35039 Marburg, Germany. Contributed to the idea, methods, analysis and critically evaluated the manuscript.; e Bas Loomans Professor, Chair of Oral Function and Restorative Dentistry, Radboud University Medical Center, Research Institute for Medical Innovation, Department of Dentistry, Philips van Leydenlaan 25, 6525EX Nijmegen, the Netherlands. Contributed to the idea, methods, analysis and critically evaluated the manuscript.; f Anne-Katrin Lührs Dentist, Hannover Medical School, Department of Conservative Dentistry, Periodontology and Preventive Dentistry, Carl-Neuberg-Straße 1, Hannover 30625, Germany. Contributed to the idea, methods, analysis and critically evaluated the manuscript.; g Nicola Scotti Professor and Dean, Dental School Lingotto, University of Via Nizza, 230 – 10126, Turin, Italy. Contributed to the idea, methods, analysis and critically evaluated the manuscript.; h Diana Wolff Medical Director of the Department of Conservative Dentistry, Medical Faculty, Heidelberg University, Im Neuenheimer Feld 400, 69117 Heidelberg, Germany. Contributed to the idea, methods, analysis and critically evaluated the manuscript.; i Reinhard Hickel Emeritus Dean of Dental School and Medical Faculty, University Hospital, Ludwig-Maximilians University of Munich, Goethestr. 70 80336 Munich Germany. Contributed to the idea, methods, analysis and critically evaluated the manuscript.; j Niek Opdam Retired Assistant Professor, Radboud University Medical Center, Department of Dentistry Nijmegen, The Netherlands. Wateringen 195, 5236 SN Den Bosch, the Netherlands. Contributed to the idea, methods, analysis and critically evaluated the manuscript.

**Keywords:** adhesive dentistry, class II restoration, composite restorations, deep margin elevation

## Abstract

**Purpose:**

There is confusion over the indications, techniques, and material choices for placing direct class II composite restorations among general dentists. This paper synthesizes new data on the general frequency of use of materials and techniques by experts, with a previous related paper that explored if and how experts restored specific class II cavities with varying complexities. It aimed to see if consensus on technique and material use when restoring class II cavities with composite could be pursued. These findings are used to inform an overview of various techniques and provide guidance on the management of more complex class II cavities.

**Methods:**

An international expert group (n = 9) developed an e-questionnaire. The first part of the questionnaire, reported here, covered the general frequency of material and technique use during cavity preparation, control of the operating field, soft tissue management, matrix selection, stabilization and peripheral seal, tooth separation, composite materials, application techniques, light curing, finishing, and polishing. It was distributed to 67 international clinicians with expertise in placing direct composite restorations (September 2024).

**Results:**

Response rate was 82%, with 95% of responders completing the first part of the survey. There was a wide variation in frequency of use of a broad variety of materials and techniques.

**Conclusions:**

International experts used a wide variety of materials and techniques for restoration of class II cavities with direct composite. Consensus was therefore not pursued. There are many potential techniques which can offer pragmatic solutions to directly restore complex class II cavities that are not beyond general practitioners.

There is a bewildering array of available materials and equipment for class II restorative procedures.^3^ UK primary care clinicians use a broad variety of them, though recommended techniques, such as rubber dam and sectional matrices, were not commonly used in a large recent survey.^4,9
^ A large majority felt that there was a lack of consensus on best practice in terms of technique and materials when placing direct alternatives to amalgam.^10^ A large majority also had low confidence when placing posterior composites with subgingival margins, which was not the case with standard cavities,^10,48
^ or when placing amalgam with subgingival margins.^10^ It is generally accepted that posterior composites are more technique-sensitive than amalgams, with a recent large international survey showing that clinicians’ main procedural difficulties during direct composite placement were keeping the cavity dry and obtaining contact points,^39^ which are less of a problem for amalgam.^4^ However, large direct posterior composite restorations can be very successful in primary care with long-term follow-up, but these restorations were often placed by experts, sometimes using multiple matrices per tooth.^4,30,31,52
^ A global commitment to phase-down and ultimately phase-out amalgam has been agreed,^66^ which means these concerns must be addressed.

While some laboratory studies can give insight into the effect of using various techniques and equipment,^7,13,19,25,26,34,44,45,51,54,55,59,63
^ the clinical relevance may be uncertain. Though much clinical trial data on posterior composite restorations exists to potentially guide a clinician on the use of appropriate materials and techniques,^1,21,27,28,43,69
^ they may be problematic in that most trials understandably tend to focus on the management of relatively simple (non-deep caries) cavities in lower-risk individuals, in an attempt to control variables.^53^ This allows the effects of changing a single variable to be seen (commonly the materials used). However, because of the generally simple nature of the cavities, attempting to broadly translate the techniques used to primary care, where cavities are often quite variable and more challenging, is questionable.^4,53
^ Such studies do not address the question of how techniques might vary to address changes in clinical variables.

Primary care data on restoration survival is more indicative of real life, where all shapes and sizes of cavities are treated, and high-risk patients aren’t excluded. Large amounts of data can be collected from general practices over long periods of time, which can be useful to understand how restorations are performing in a population of interest.^33,36,42,46,52,53,61,65
^ It has been shown that the various composite materials generally only have minimal effects on outcomes, and the cavity size, presence of a root filling, operator, and risk status of the patient are often more important, for example.^15^ Varying outcomes among operators is related to multiple variables.^37^ The patient base and their levels of disease and risk are important, but variation in skill and selection of appropriate techniques for the given clinical presentation are also likely significant factors. Outcomes in non-trial datasets are also likely influenced by indication bias, where operators intervene at different times and in different ways (eg, when choosing to place a specific material in a specific situation, or direct or indirect restoration),^4,33,35,36,38,68
^ which may be influenced by the availability of appropriate materials or equipment in a practice.^17,57
^ There is likely still much variation in treatment planning among dentists, judging by recent evidence,^12,18,64,68
^ which can also be influenced by patients and their varying values, including potential financial restrictions and the structure of the overarching health system, for example.^4,8
^ Clinical guidance may also vary between different bodies, potentially creating confusion.^16^


Basic data on how various restorative interventions affect tooth loss at the population level over the long term^46^ and more in-depth, but short-term data are available.^37^ However, they provide very little insight on how variation in presentation of a class II cavity or treatment planning affects tooth retention, because the variable presentations at intervention are often not fully controlled for and can affect management.^4,6,7,53,65
^ The data collection burden, in terms of both numbers of cases and variables, that would be required to allow better understanding of the important factors relating to treatment planning, use of equipment and techniques and how they relate to outcomes in varying clinical situations is enormous. These issues make the currently available primary care data inappropriate to provide guidance on class II restorative technique and decision-making in different clinical situations.

Translating currently available clinical and laboratory data on class II composite restorations to guidance on clinical techniques as cavities vary is therefore difficult. There is minimal guidance for practitioners on dealing with varying clinical presentations, which has been recognized by primary care clinicians. This likely contributes to a lack of confidence in placing posterior composites in difficult situations, and variation in treatment planning. The objectives of this study were therefore to quantify general variations in the use of techniques, equipment, and materials by experts for the direct restoration of class II cavities with composite. Allied with findings from a previous related study, which explored the willingness of experts to directly restore specific class II cavities and the techniques they would use,^6^ the aim was to see if experts could reach some consensus (with acceptable variations) on direct restoration of class II cavities with composite. An exploration of the use of some more novel, non-standard techniques and demonstration of their application through case presentations would also be included. This would then lead to guidance on the use of techniques to restore more complex presentations of class II cavities with composite.

## Methods

An expert panel of nine restorative dentists from five countries (Germany, Netherlands, Italy, Switzerland, UK), with an interest in direct restorative dentistry, initially met online to develop a project to investigate the use of different techniques and materials to restore class II cavities. An associated project exploring willingness to restore specific presentations of class II cavities and materials and techniques used has been published elsewhere.^6^ Panel discussions led to the development of a questionnaire, the first section of which explored the general frequency of use of materials and techniques for class II composite restorations and will be described in this paper. The panel piloted the survey, providing feedback, and this was collated and used as a basis for discussion at three in-person workshops at the combined European Federation of Conservative Dentistry (EFCD) and Organization for Caries Research (ORCA) Conference, July 2023, in the Netherlands. Changes were made to the wording and structure of the survey, and aims and objectives were defined. The survey was repiloted to the expert panel prior to distribution, but no further changes were suggested.

The study is reported in line with STROBE (Strengthening the Reporting of Observational Studies in Epidemiology) guidance and a favorable ethical opinion was obtained from Newcastle University Research Ethics Committee (ref: 30898/2022). The e-questionnaire was developed using SurveyMonkey software and asked respondents to indicate their general frequency of use of various materials and techniques when restoring class II cavities in Section 1 (reported here). The frequency options were: always (100%); commonly (< 100–50%); sometimes (< 50–10%); rarely (< 10% > 0%); or never (0%). These questions were supplemented by further specific questions. The questions related to cavity preparation, control of the operating field, soft tissue management, matrix selection, stabilization and peripheral seal, tooth separation, composite material types and application techniques, light curing, and finishing and polishing, ordered as a restoration would be completed. Free text comments were allowed at the end of each section, and a PDF version of the survey instrument is included in Supplementary Information 1.

The survey was distributed using a closed sampling frame to a convenience sample of 67 experts known to the panel by personal e-mail on September 18, 2024. Two reminders were sent, and the survey closed on November 8, 2024. Further details of the methods, including survey development, sample selection, and distribution alongside respondent demographic information, have been previously described.^6^


Responses were exported from SurveyMonkey into an Excel spreadsheet and anonymously forwarded to the research team, where they were cleaned and Section 1 analyzed in Stata software (version 18; StataCorp MP) with descriptive statistics.

## Results

Response rate was 82%, with 95% of responders completing Section 1 of the survey reported here. Demographic information has been reported elsewhere.^6^


As an example, responses relating to the frequency of use of various cavity preparation techniques are shown in Figure 1. Though use of diamond burs were almost always used by all respondents, use of tungsten carbide burs, airborne particle abrasion, oscillating or ultrasonic tips, and barrier protection of the adjacent tooth were very variable. Fifty-five percent (55%) always cleared all proximal contacts. Fifteen percent (15%) never beveled cavity margins, and 15% always beveled enamel cavity margins. Thirty-two percent (32%) preferred a box-like cavity preparation outline, 32% a flared outline, and 36% had no preference.

**Fig 1 fig1:**
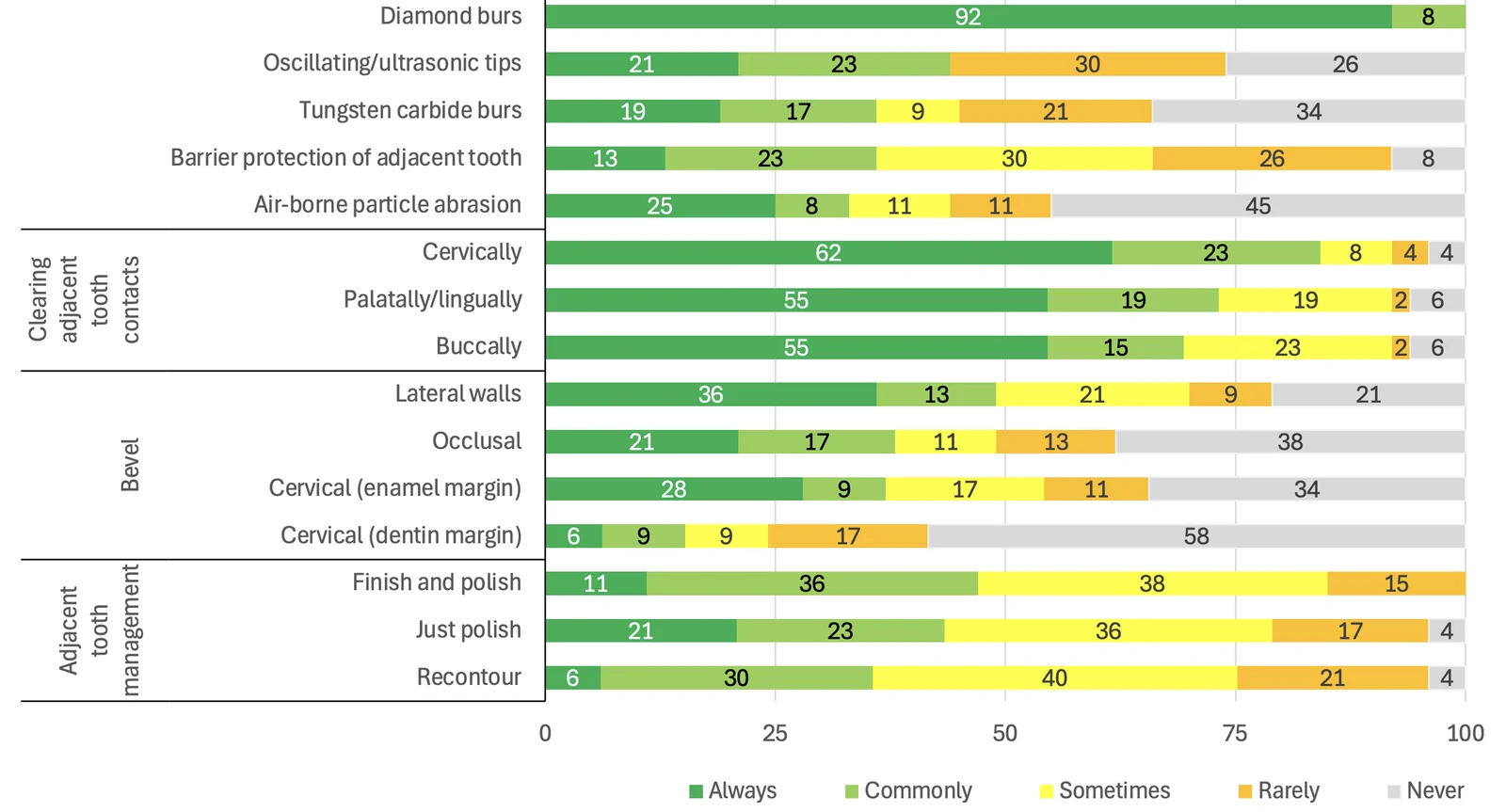
Experts’ frequency of use of various cavity preparation techniques.

The frequency of technique and material use for control of the operating field, matrix selection, techniques used to obtain desired restoration shape (including when cusps were missing), matrix stabilization and peripheral seal, tooth separation, composite material, application techniques, finishing and polishing, and respondent preference on matrix band thickness are shown in Figures 2 to 11. It can be seen that, in general, all of the materials and techniques asked about were used with varying frequencies. The vast majority had some responses of “never” and “always.” The exceptions in the “always” category (0% responses) were generally quite niche or unusual techniques which were perhaps rarely used and only indicated in more challenging cases, for example, direct shaping by occlusion (DSO) where the patient bites into unset composite to guide occlusal form, the use of an Elliott Separator for tooth separation, or the Teflon Floss technique for matrix stabilization and peripheral seal.^3,55
^


**Fig 2 Fig2:**
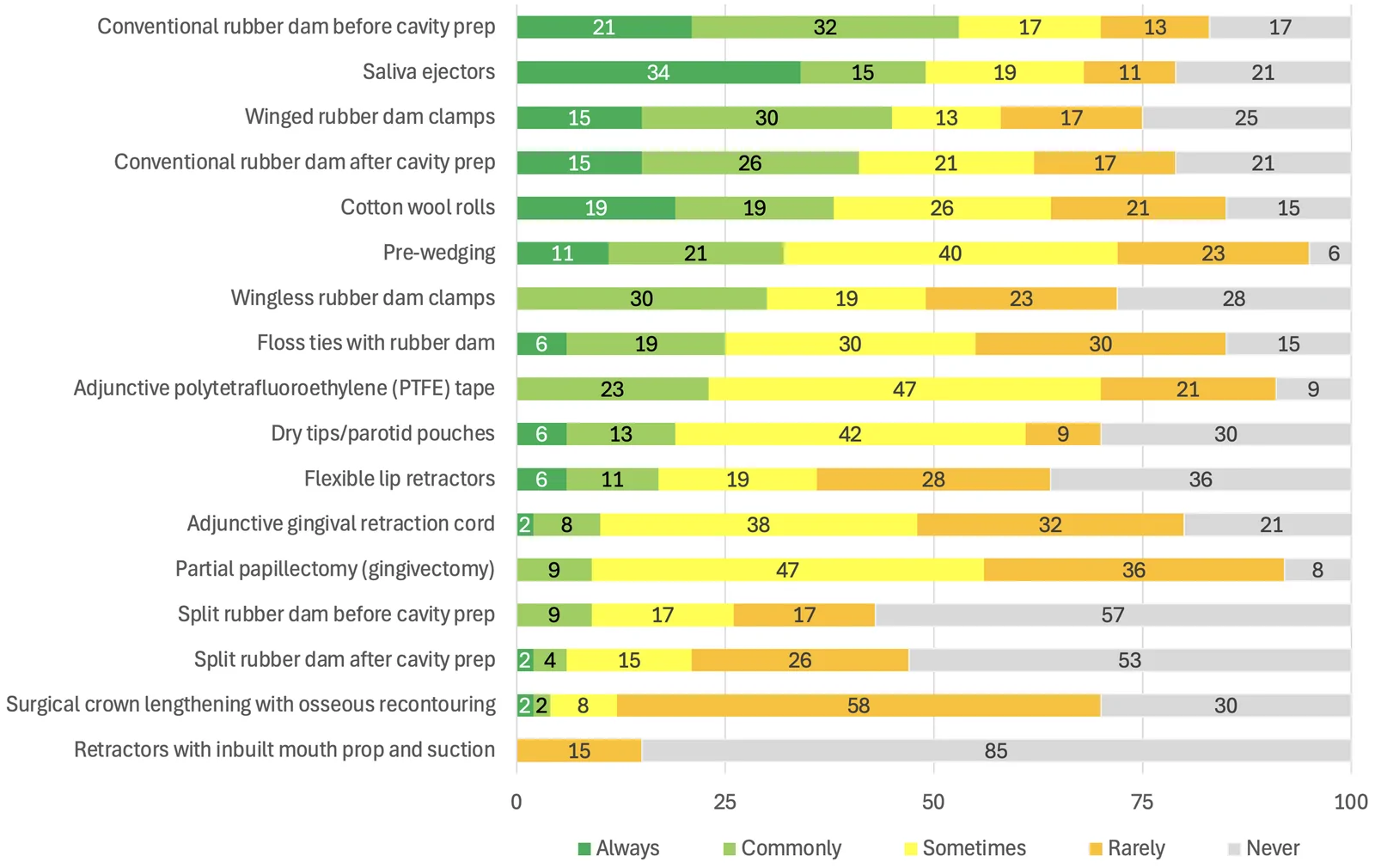
Experts’ frequency of use of methods to control the operating field. Other responses specified: liquid dam (3), hemostatic agents (3), vasoconstrictor-containing local anesthetic injection (1), air drying (1).

**Fig 3 Fig3:**
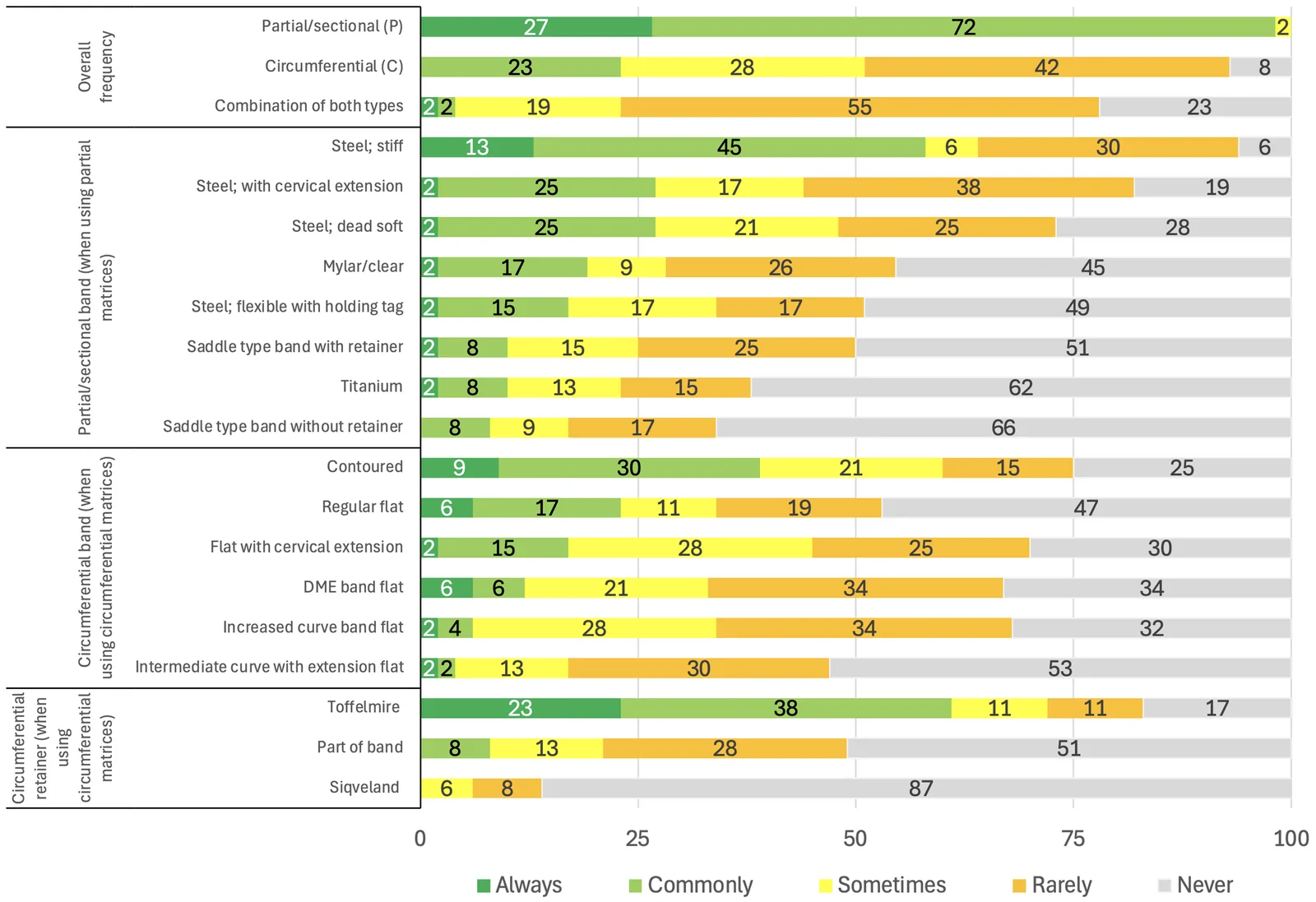
Experts’ frequency of use of matrices (DME = deep margin elevation). Other responses specified: specific circumferential matrices with retainer included (4) – these were intended to be captured as “circumferential retainer as part of band.” Customization of circumferential band (4) and customization of sectional to avoid interference with rubber dam clamp (1).

**Fig 4 Fig4:**
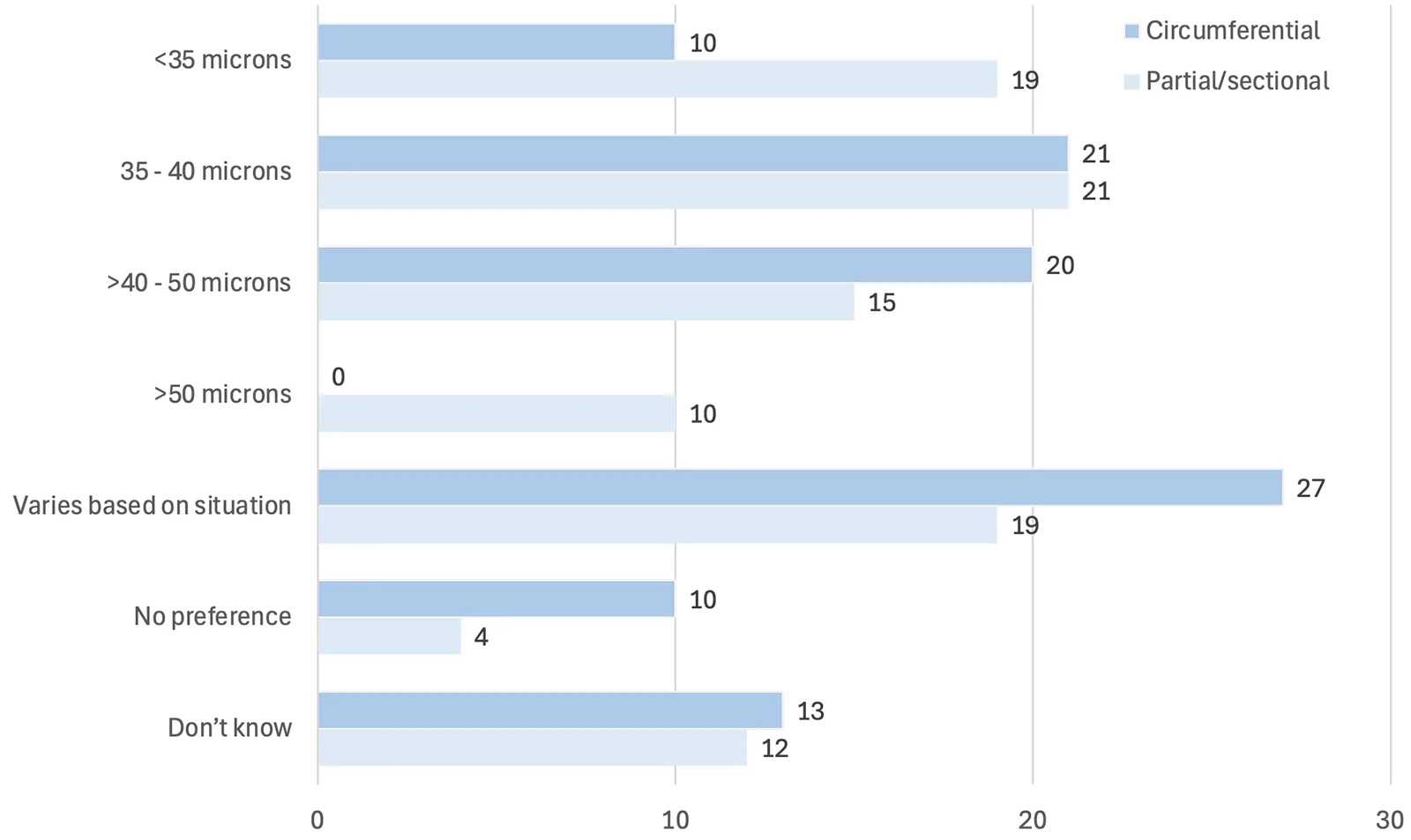
Matrix band thickness preferences. Explanation of choice: comments relating to stiffer is better (6), reducing deformation (6), relating to mylar – needs to be thicker, 75 micron (3).

**Fig 5 Fig5:**
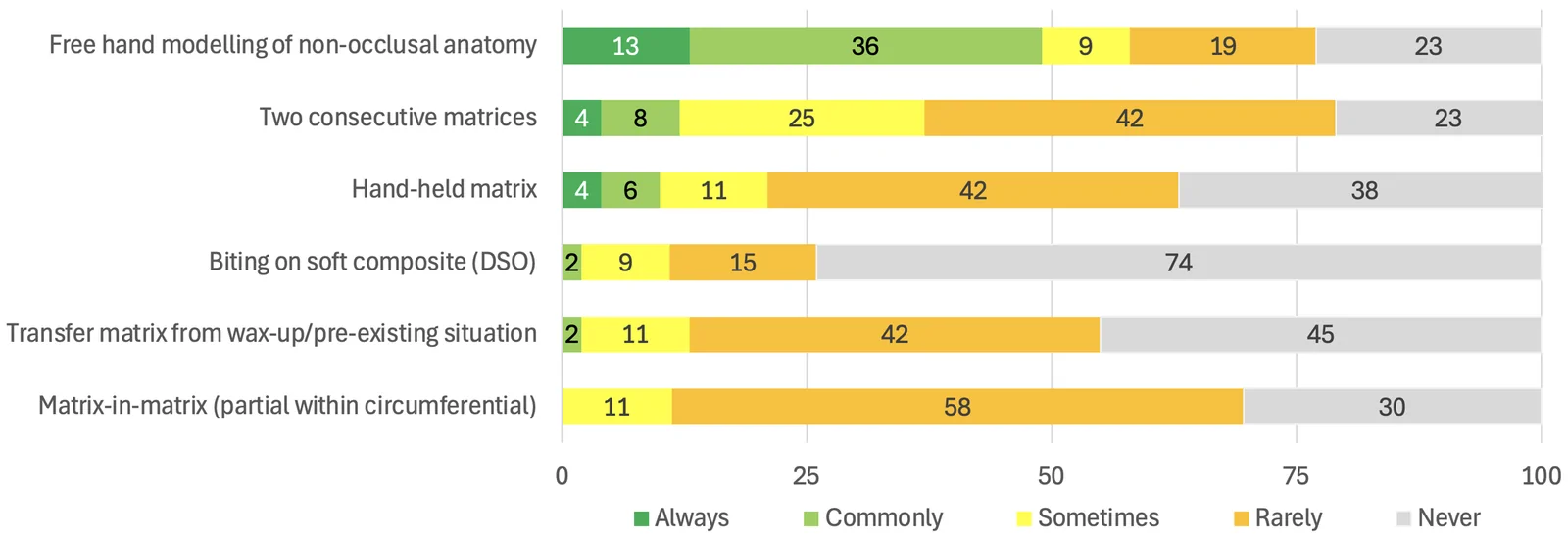
Experts’ frequency of use of techniques to obtain desired class II restoration shape. (DSO = direct shaping by occlusion.) Other responses specified: specific mylar contoured matrix push-pull technique (1), overfilling and bur contour (3).

**Fig 6 Fig6:**
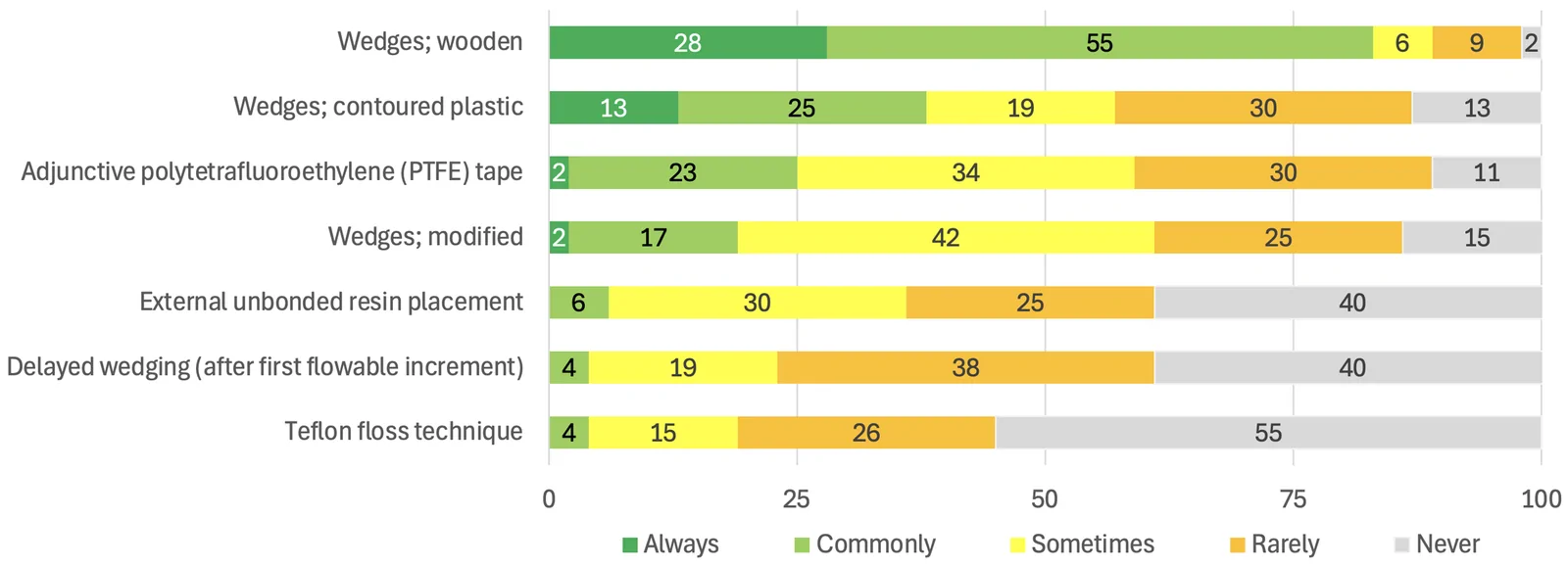
Experts’ frequency of use of matrix stabilization and peripheral seal techniques. Other responses specified: External application of liquid rubber dam or temporary inlay material, which are both commonly resin-based (2). Delayed placement of separation rings (1).

**Fig 7 Fig7:**
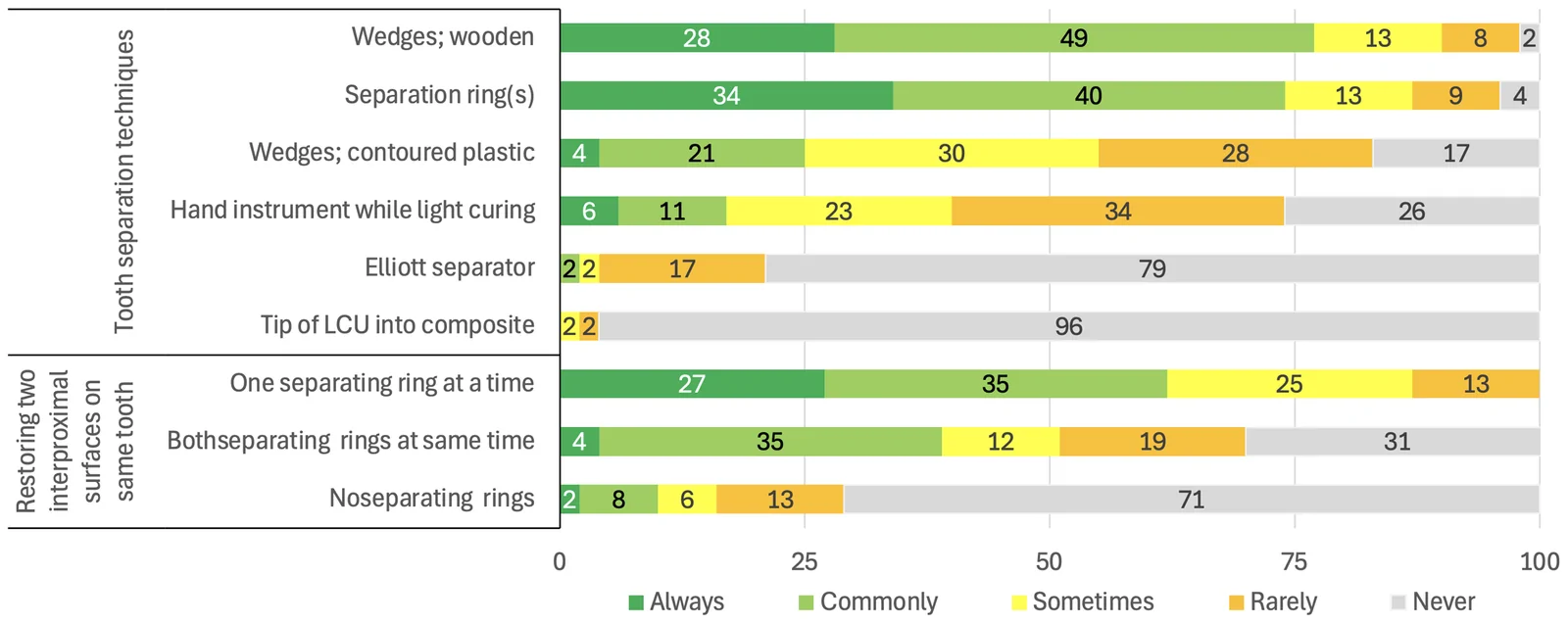
Experts’ frequency of use of tooth separation techniques. Other responses specified: Elliott ring (2); specific hand instruments named (3).

**Fig 8 Fig8:**
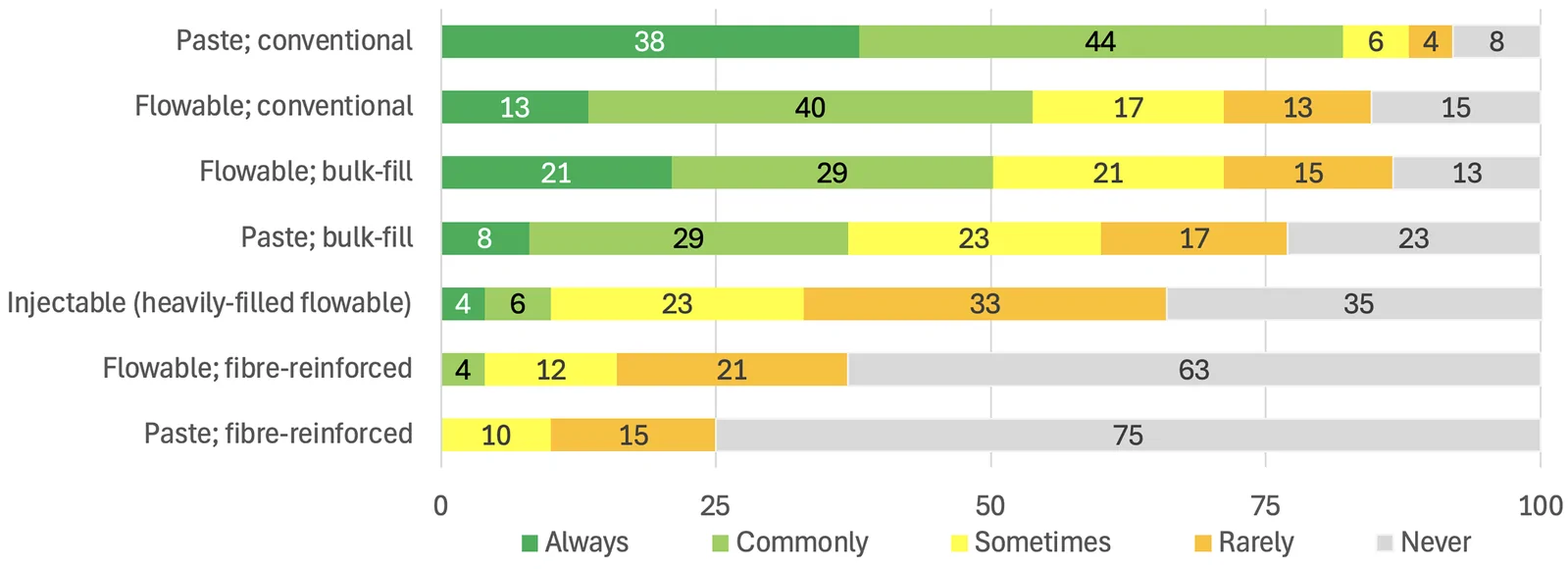
Experts’ frequency of use of composite materials. Other responses specified: dual cure (when will be used as core) (1).

**Fig 9 Fig9:**
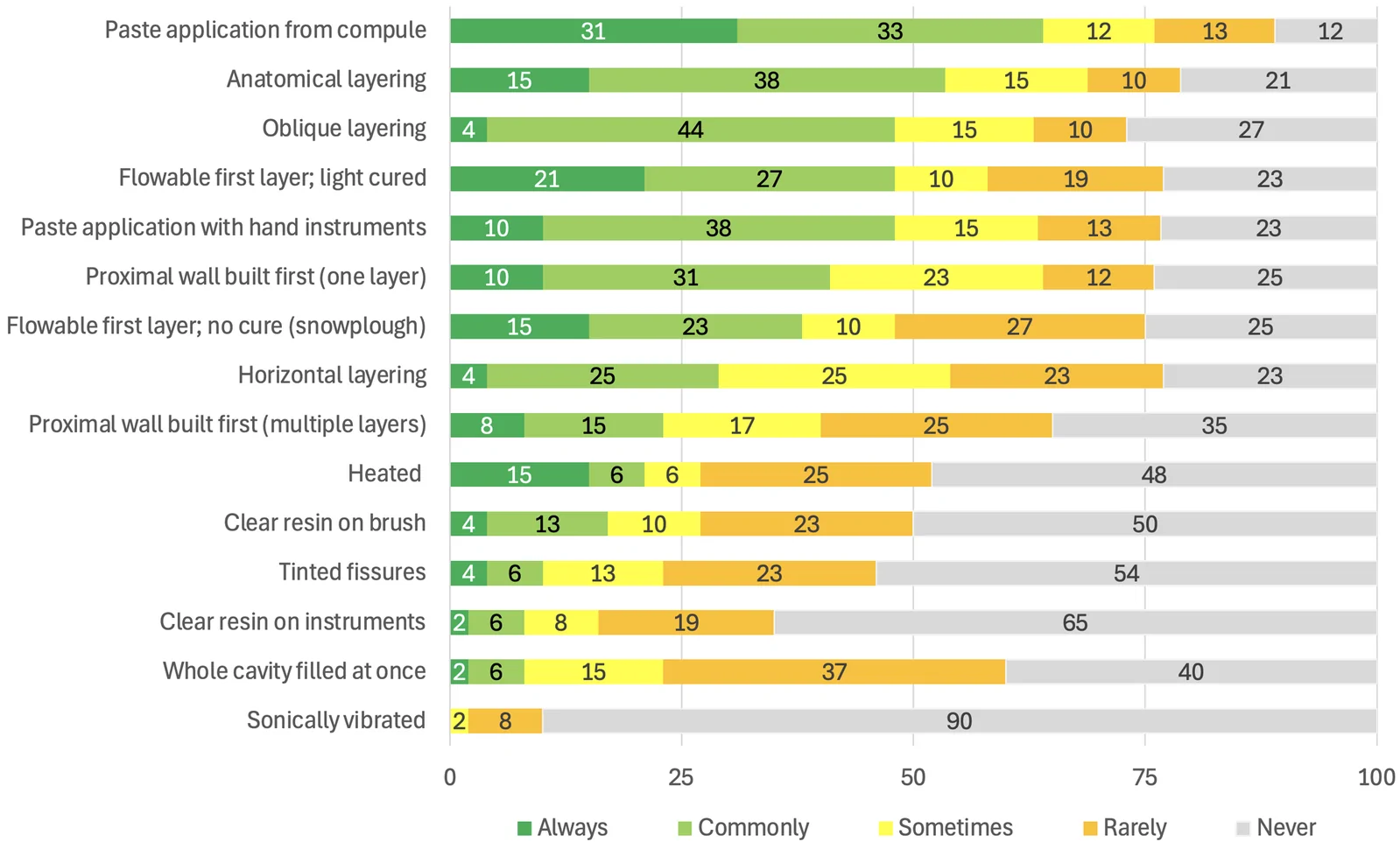
Experts’ frequency of use of composite application techniques. Other responses specified: modeling liquid with brush (1) – clear resin on brush was an option.

**Fig 10 Fig10:**
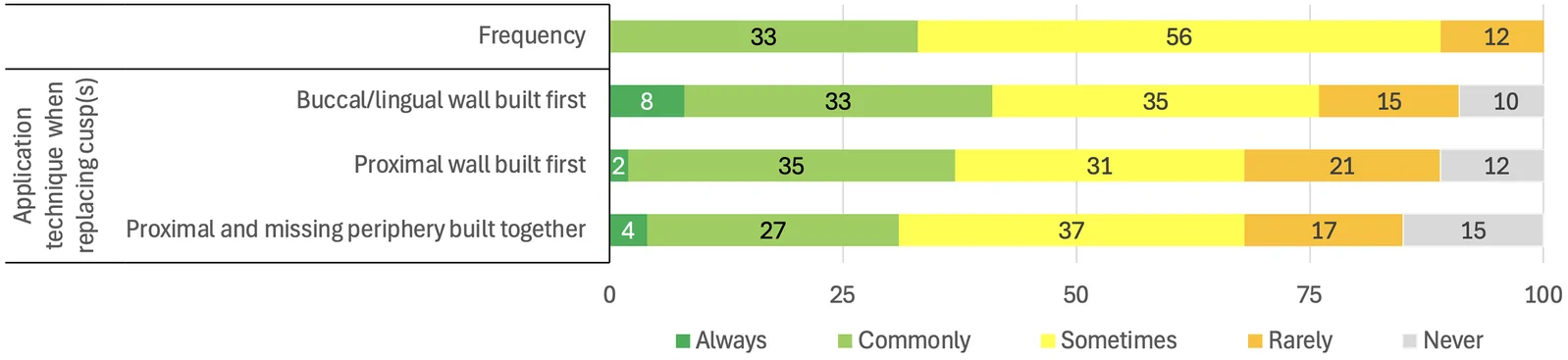
Experts’ frequency of placement of cusp-replacing restorations. Other responses specified: injection-molding (snowplow) (2) after stabilization of matrices with resin (1).

**Fig 11 Fig11:**
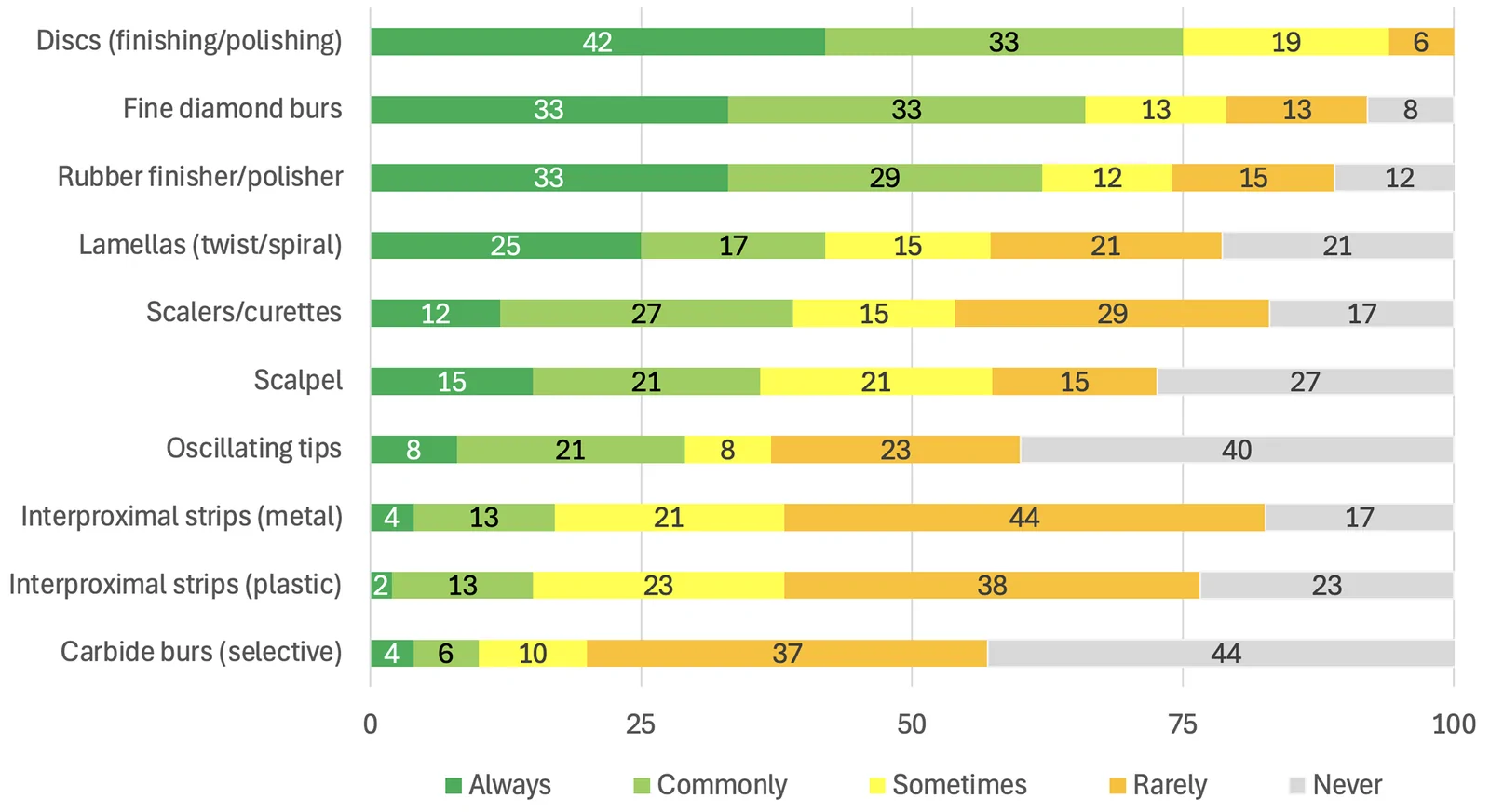
Experts’ frequency of use of finishing and polishing techniques. Other responses specified: occlusal polishing brush (3); stones (2); polishing paste (2); nylon bristle brush (1).

A majority used a multiple peak light curing unit (LCU) with an LED tip and a continuous radiant emittance of 1000–2000 mW/cm^2^ for longer than recommended in the material instructions. A majority (79%) used additional exposure from buccal or lingual directions, though a minority (15%) used two LCUs simultaneously. Thirty-one percent (31%) gave a final cure under air-block (eg, glycerin).

No one used just one manufacturer of matrix bands (circumferential and partial/sectional), wedges, and rings. 25% used more than one circumferential band manufacturer, 33% used more than one partial/sectional band manufacturer, 29% used more than one wedge manufacturer, and 44% used more than one ring manufacturer.

## Discussion

Experts used a broad variety of materials, equipment, and techniques at each step in the process of directly restoring class II cavities with composite, and the approach varies based on the cavity presentation. Given these results, and following the presentation of the data analysis to the expert group at CONSEURO25 (April 2025 in Spain), a verbal consensus was reached to not pursue a consensus. It is clear that there are many acceptable paths to providing successful restorations, and the cavity presentation influences this. If clinical situations increase in complexity, the requirement for a broader range of procedures and techniques correspondingly rises, as demonstrated by the survey data.

It was therefore deemed more appropriate to provide guidance on class II composite placement, drawing on the data obtained from the survey as a whole.^6^ This will highlight some of the less commonly used techniques which commonly aren’t seen in textbooks and have been identified as being more commonly used in more complex cases by experts. This will involve case examples, including a rationale for the use of the techniques shown. The focus will be on matrix selection and stabilization, tooth separation and composite application techniques. Previously published step-by-step guidance on techniques will also be highlighted for readers to refer to.

Matrices can be stiff, flexible or “dead soft”, and metal or clear Mylar. They come in a variety of shapes, emergence profiles, sizes, and thicknesses. All of these were used by the experts surveyed, with their differing properties often leveraged in different clinical situations. The thinner the matrix, the less tooth separation is required to obtain a contact, but the less rigid the matrix is, increasing the chance of band flattening on tightening with circumferential matrices and distortion of both circumferential and sectional matrices. A majority of experts preferred a rigid metal matrix, but a significant proportion used malleable matrices, which are more easily burnished. This can help to form a contact, but has a greater chance of distorting the matrix, which can lead to rougher restoration surfaces that may be more difficult to clean. Clear Mylar bands generally have to be made thicker to allow them to be placed without distorting and therefore require more tooth separation to achieve a contact.^3^ However, composite can be cured through them, reducing the potential for uncured material in the base of the cavity compared to metal matrices. Plastic wedges are commonly hollow, having a low profile, which allows them to engage and seal matrices subgingivally without impeding the emergence of the matrix, which could prevent the formation of a contact point. There is, however, more potential for them to fail to engage a matrix, and their potential to separate teeth is uncertain.^3,7
^ Wooden wedges are better at engaging matrices and have the potential to predictably separate teeth sufficiently to obtain a contact for matrices ≤ 50microns thickness.^3,29
^ They are, however, less likely to be able to engage a matrix subgingivally, with more potential to distort a matrix on placement. Wooden wedges are also taller than hollow plastic wedges, so they can potentially prevent a matrix from forming a contact point.^5^ This can be mitigated by adjusting the wedge,^5^ or using both wedges sequentially, utilizing their specific properties in tandem. Separating rings of various designs were preferred by different respondents and can be used to coronally stabilize matrices and provide excellent tooth separation.^43,63,69
^ They can, however, cause a variety of distortions which occur unpredictably^3,7,13,60
^ and rings can be difficult to apply where boxes are broad, or cusps are missing, without taking prior steps, for example by using multiple matrices in two (or more) stage techniques.^24,41,47
^ The reader is pointed to alternative texts for more thorough consideration and guidance on the use of sectional matrices, wedges, papillectomies and separation requirements and techniques.^3,5,24,41,47,60
^


Data from the first paper also show that when present, cavity complexifying factors affect materials and techniques used.^6^ Deep subgingival cavity margins were much more frequently managed using papillectomies, which can allow effective isolation, matrix and wedge placement in subgingival cavities,^5^ as were more curved circumferential matrices which are used in Tofflemire retainers. The extra flare on these matrices can seal deep margins, often without the absolute need for a wedge or rubber dam, as they are actively tightened when placed, and can also build more aggressive emergence profiles which facilitate establishment of contact points or areas (Fig 12). The extra curve is helpful in simple one-band and more complex two-stage (or more) techniques, as described and shown schematically in Figure 13, and shown in case examples in Figures 14, 15 and 16.

**Fig 12a to f Fig12atof:**
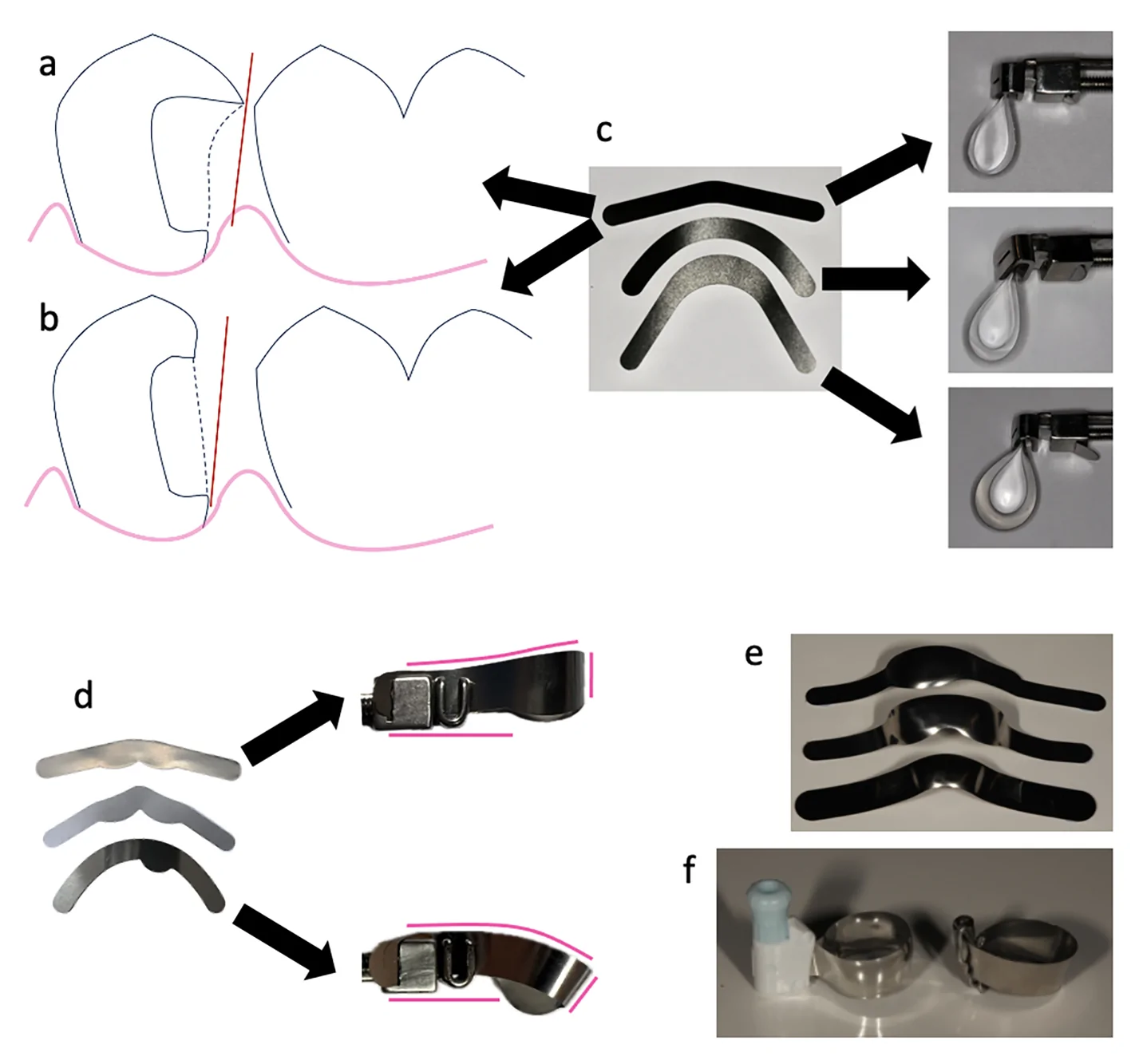
Common problems seen with classic, small/no curve matrix bands: they struggle to seal the cervical margin in deep cavities where a lateral wall extends close to the adjacent tooth (a), or are able to seal the margin but cannot form a contact where broader boxes are present (b). Circumferential matrices come in a variety of sizes and shapes. Increasing the matrix curvature increases the flare (c). Extensions help to seal deep interproximal cavities, and increasing the curve facilitates deep subgingival placement unimpeded by the retainer (d). Contoured circumferential bands are available with and without extensions (e), alongside those which do not require a conventional retainer (f). The open U part of the retainer is classically placed downwards toward the gingiva, as shown in Figure 12c, which provides a tight cervical seal. However, if the U part is placed the opposite way around, as shown in Figure 12d, the matrix will flare more coronally, aiding the formation of contacts and will tend to reach slightly further subgingivally. It also tends to be more stable, especially where the wall which the head of the retainer is engaging is sloping toward the center of the tooth, as is commonly seen on the buccal walls of mandibular molar teeth, for example, and resists the matrix lifting off the tooth.

**Fig 13a to i Fig13atoi:**
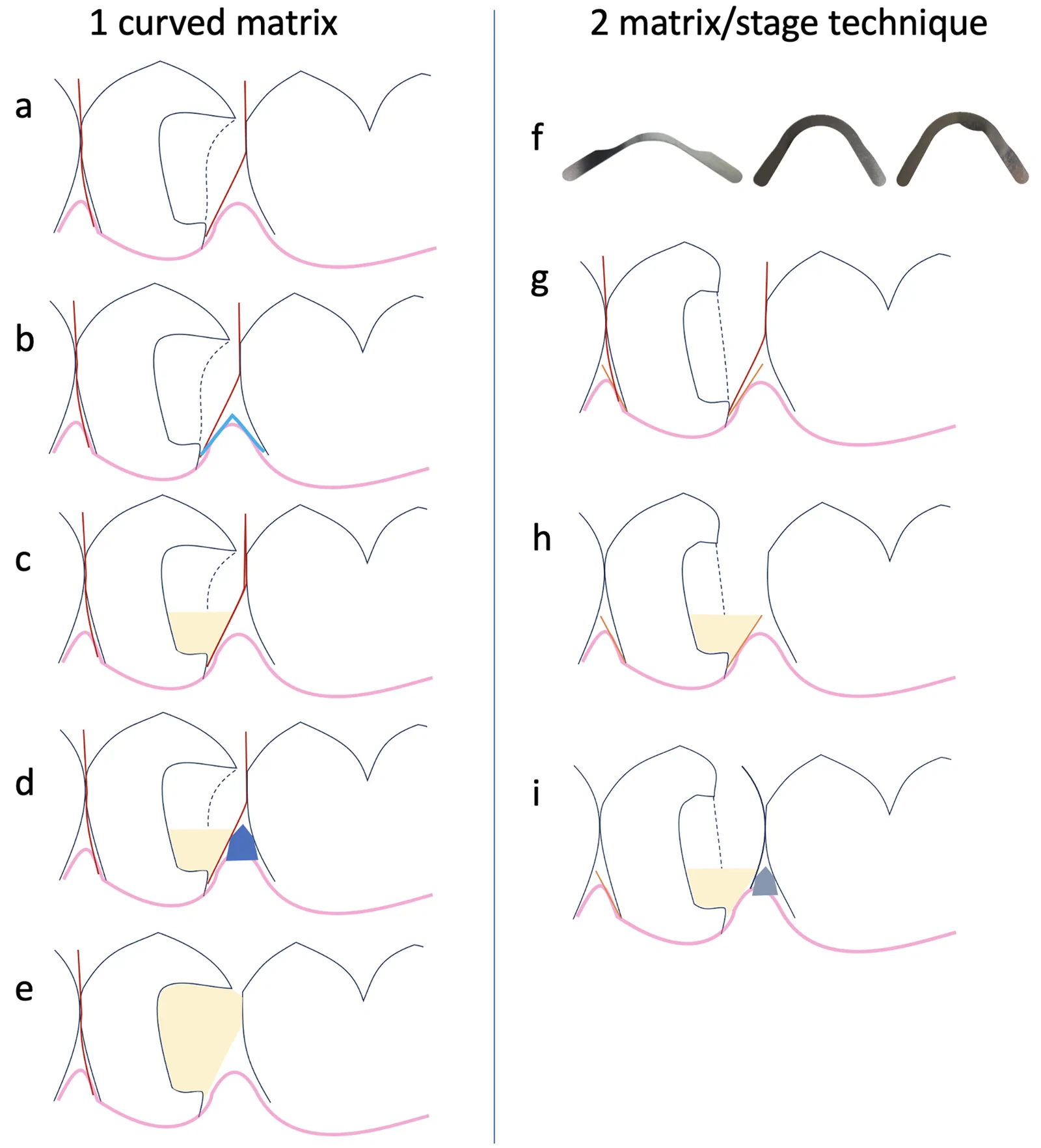
One matrix and two matrix/stage techniques using increased curve circumferential matrix bands. Increasing the circumferential matrix curvature helps the matrix to seal cervically when tightened and also form contacts across larger bridging gaps due to the increased flare (a). Low profile plastic wedges may help form a seal, but are commonly not necessary (b). A “hip” of composite can be built (c), which then allows a progressive or delayed wedging technique. A wooden wedge can now be firmly placed without risk of deformation of the matrix to achieve separation of the teeth more than the thickness of the matrix (d), or a separation ring could now be placed. Subsequent increments of composite can then be placed, allowing the formation of a contact when the matrix is removed (e). Narrower bands are available in varying curvatures and with extensions (f) which can be placed through and beyond the contact point(s), which allow even greater flare than standard curved bands, which are limited by the contact on both sides (g) (Alternatively, regular curved bands can be trimmed to the required shape). They allow placement of a “hip” of composite with a more aggressive emergence profile to raise the restorative margin and shift it as far as possible toward the adjacent tooth, facilitating treatment of large bridging gaps (h). (All but the largest of bridging gaps can, however, be treated with conventional, untrimmed curved bands to build a hip in two-stage techniques.) This is commonly termed a “deep margin elevation,” especially when performed prior to indirect adhesive restorations, but can be used for multi-stage direct restorations. Standard sectional matrices and wedges can then be used to form the contact (i). The two-stage technique is more prone to ledges at the interface of the hip and subsequent increments and has an increased chance of contamination relative to the single-stage technique, but can treat cavities with larger bridging gaps and provide more idealized interproximal form and contacts.

**Fig 14a to h Fig14atoh:**
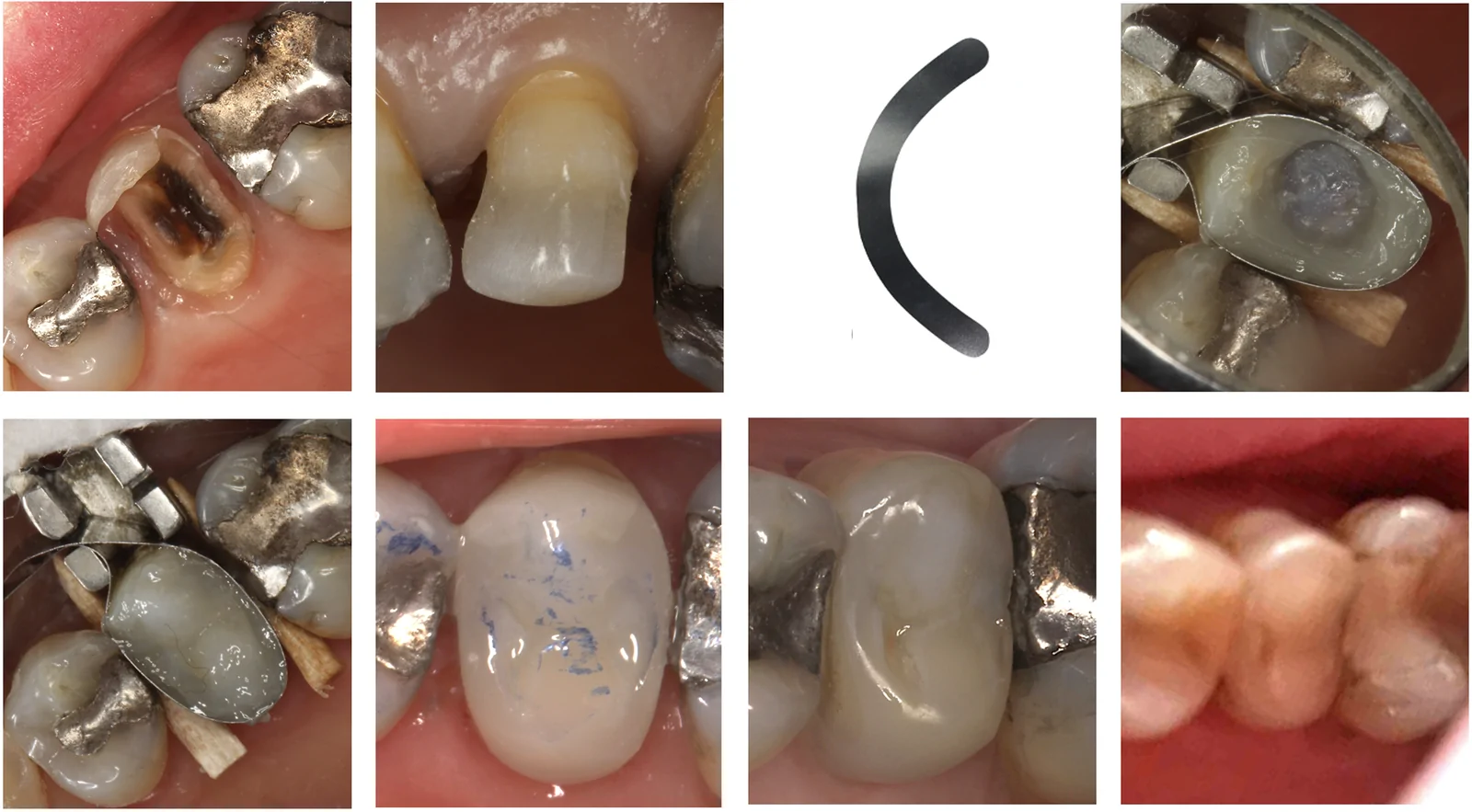
Simple one-stage curved circumferential matrix case. The cavity had deep subgingival dentin margins around the majority of the tooth, a large bridging gap, broad boxes with mesio-occluso-disto-palatal extension, which were shown to be complexifying factors,^6^ and a thin remaining buccal cusp (a). It was treated in private general practice. Treatment options and their prognosis were discussed with the patient, and they accepted the costs and risks of performing a direct composite restoration. It was restored simply with cuspal reduction (b), an increased-curve circumferential matrix band (c) (40 micron) in a one-stage technique, relative isolation, and a mix of conventional paste and bulk-fill flowable composites, with the periphery built using a snowplow technique all at once (d to f). Firmly placed wooden wedges achieved the required separation to achieve contacts with adjacent teeth. A 6-year follow-up is shown (g), and a photo of the tooth was sent by the patient showing survival at just under 14 years (h).

This is especially so where bridging gaps are large, as shown by data correlating their use with these cavities in the previous study.^6^ Tooth separation to achieve contact should be considered and obtained later in the procedure, however. An example of the use of a curved circumferential matrix alone is shown in Figure 14 to restore a cavity which only 27% of experts were willing to restore with direct composite.^6^ It follows a simple one-band curved matrix approach as shown in Figure 13 and serves to show an example of how a restoration in a complex cavity with guarded prognosis can be successful with simple techniques, but perhaps rarely available equipment (the matrix band), which would not have been attempted by a large majority of expert respondents in this survey. Fairly large retrospective clinical data have shown that large, complex direct composite restorations can be successful over the long-term, as has a long-term randomized controlled trial with or without the use of rubber dam.^30,31,52
^ Equally, there is limited evidence showing no obvious differences in outcomes when comparing direct and indirect composite restorations in vital teeth and direct composite with indirect restorations in non-vital teeth,^14,32
^ and S3 German clinical guidance advocates direct composite for cusp replacement restorations.^70^


Obtaining a robust seal at the base of a deep subgingival cavity with sectional matrices can be more difficult as they are commonly passively placed without a retainer. It is however possible and can be helped by tucking the matrix into the rubber dam, which can also be helpful in creating a seal, as shown in Figures 15 and 17. Flexible clear matrices can lend themselves to this approach. Saddle matrices, which are often taller and more contoured than regular sectional metal matrices, can combine a sectional matrix with a retainer as shown in Figure 17, or be used without the retainer, as shown in Figure 15. Polytetrafluoroethylene (PTFE) tape can also be used adjunctively to help obtain a seal as shown in Figure 17, and unbonded flowable resin can be placed external to matrices to stabilize them coronally against adjacent teeth or matrices.^3,7
^ However, sectional matrices are not especially stable when placed in these ways; therefore, using a first increment of flowable composite which is then cured was also more likely to be employed for deep subgingival cavity margins by experts, as described in Figure 15. This is likely in an attempt to take advantage of the flow characteristics to obtain a more passive adaptation of the material, which may avoid opening a gap at the periphery of the cavity. Packing paste composite firmly to ensure adaptation to the cavity and elimination of voids risks opening gaps and extrusion of material beyond the margin; however, using heated paste composite (which increases the flowability of the material) can potentially overcome these concerns, as shown in Figure 17. Increased curve or deep margin elevation type circumferential matrices are therefore often used to build the initial hip (or wall), as the retainer allows the band to be tightened firmly, creating a more robust seal, as shown in Figures 13, 15 and 16. This enables the application of the snow-plow or injection molding composite application technique (which involves injecting paste composite under pressure from a compule into previously placed, but uncured, flowable composite) with minimal risk of extrusion.^51^


**Fig 15a to l Fig15atol:**
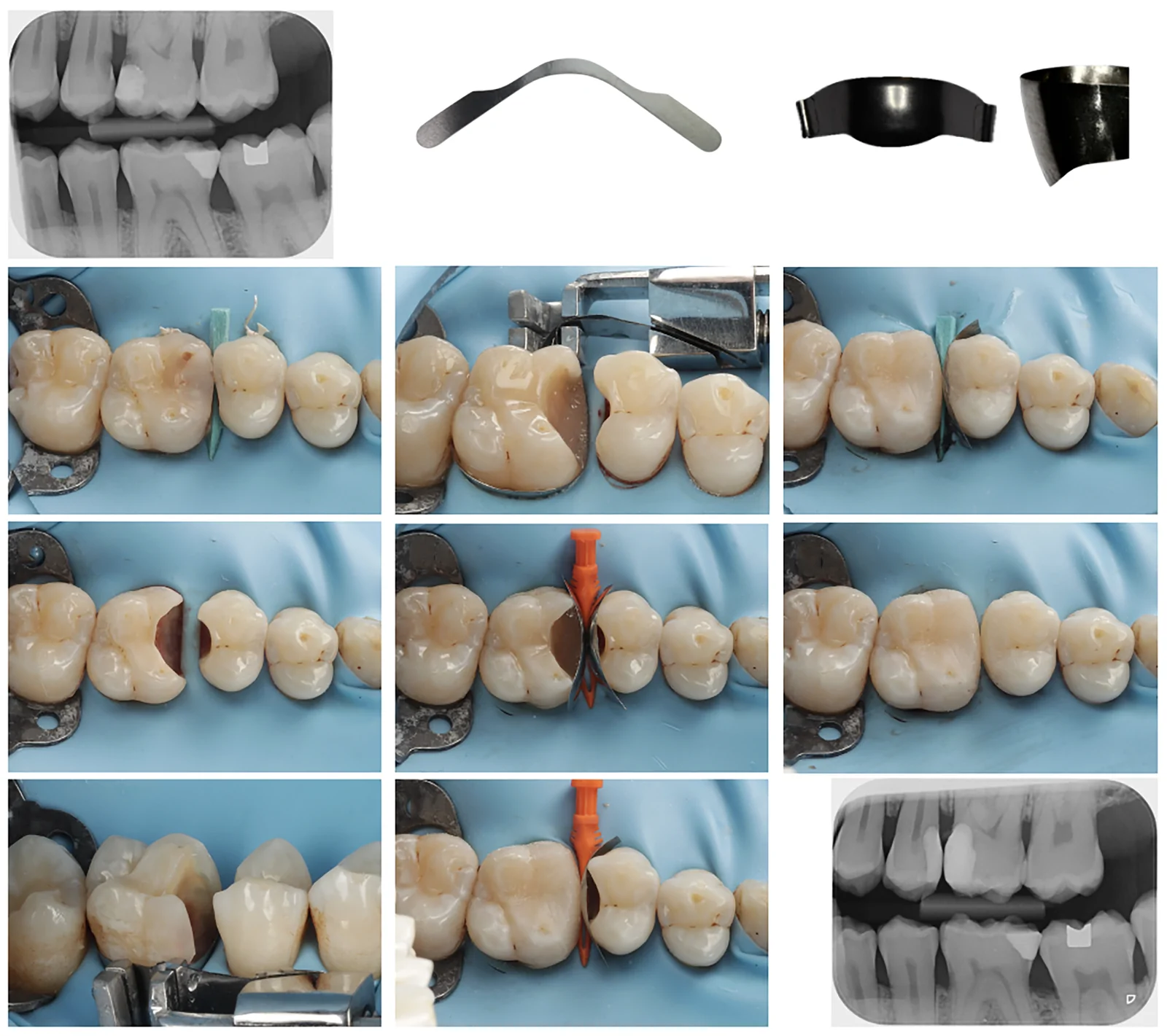
Management of complex adjacent deep subgingival cavities. Case courtesy of Alan Burgin. Preoperative bitewing radiograph showing deep subgingival, sub-cemento-enamel junction caries 25 distal and 26 mesial (a). Rubber dam, floss ligatures, and pre-wedging applied prior to cavity preparation to retract and displace the papillae (b). Deeper molar cavity managed with a two-stage technique, first stage using a 50-micron, stiff curved deep margin elevation (DME) circumferential matrix band to raise the restorative margin using a snow-plow application technique (c to f). (g) The second stage involved placement of a standard stiff metal 50-micron sectional matrix band to restore the molar with a raised margin, and the premolar matrix was placed at the same time to prevent the molar restoration from impinging on the premolar restorative space. It was not restored at the same time, however. The matrix was stabilized by tucking it under the rubber dam, and a plastic low-profile wedge was placed to aid adaptation at the cervical margin of 26. No tooth separation was required at this stage, as contact was not being formed. Molar restored by building interproximal wall first with a snow-plow technique prior to building occlusal anatomy (not shown), and molar matrix removed (h). (h and i) Premolar managed with single tall trimmed stiff 50-micron saddle matrix again tucked under rubber dam to stabilize. Plastic contoured wedge allows subgingival seal to be obtained, but an uncertain amount of separation, which cannot therefore predictably create a contact. A wooden wedge would not seal subgingivally here and would also potentially extend too far coronally, preventing the formation of a contact or distorting the matrix. Initial increment of flowable composite placed and light-cured to create a “hip,” as the matrix is well adapted but not especially robust. (j) Plastic wedge removed, and largest wooden wedge that could be inserted driven in to engage hip and provide tooth separation to facilitate contact formation using a sequential wedging technique. (This is similar to the schematic diagrams shown in Figure 2, but with the use of a tall sectional, rather than a curved circumferential matrix). No separating ring was placed to avoid matrix deformation, and further separation was not required. The snow-plow technique with bulk-fill flowable and paste composites together was then used to restore the remainder of the cavity in one increment, as there was insufficient space to build the proximal wall first. (k and l) Removal of matrix shows contact formation, and the radiograph shows acceptable restoration margins and emergence profiles.

**Fig 17a to i Fig17atoi:**
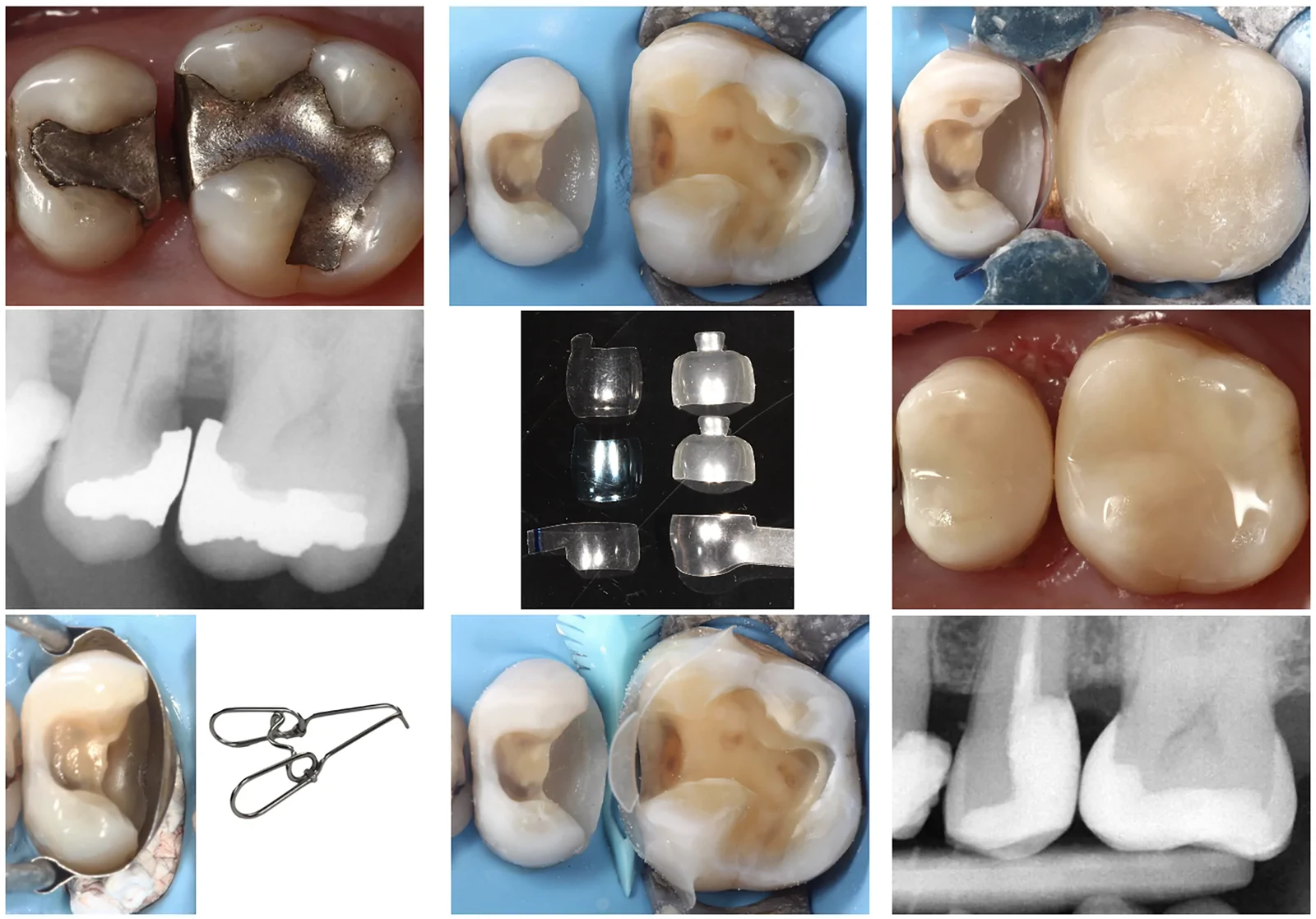
Management of complex adjacent subgingival cavities. Case courtesy of Kazhan Mohammed. (a and b) Preoperative situation, extensive recurrent subgingival, sub-cemento-enamel junction caries 25 distal and ledged restoration 26 mesial with open contact in between and a large interproximal bridging gap. (c) Papillectomy performed (not shown) to allow sealing of rubber dam on deep 25 margin. Saddle matrix tucked under rubber dam and used with spring clip to stabilize, as there is no adjacent contact. Adjunctive PTFE placed to obtain seal as a wedge cannot be effectively placed here. (d) Hip (deep margin elevation) built with heated paste composite injected into initially placed but uncured flowable composite using a snowplow technique as part of a two-stage technique. In this case, it was also used as a pre-endodontic build-up to allow placement of a robust, well-sealed rubber dam to facilitate effective and safe disinfection of the root canal system with irrigant. Root canal treatment was completed and the access sealed. (e) Clear matrices come in a range of shapes and sizes with variable contour and wrap. (f) An aggressively contoured clear matrix was used to deal with the large bridging gap when restoring 26. It sealed below the base of the cavity by being tucked into the rubber dam to stabilize it, alongside the additional use of a low-profile contoured plastic wedge (as shown in Fig 2), allowing an “overmold” of composite beyond the cervical extent of the cavity. This created a smooth, cleanable emergence profile. No contact is being formed at this stage; therefore, there are no separation requirements. Note that the 26 is the most distal tooth in the arch and therefore needs to be clamped (to use rubber dam) and restored at the same time. A wingless clamp was used to facilitate access to the interproximal area to aid with matrix placement and wedging. Winged clamps can sometimes get in the way of these processes. (g) A clear matrix with less aggressive contour than the previous matrix was used to obtain an appropriate contact because the bridging gap was now relatively smaller. A modified wooden wedge and separating ring were used to stabilize the matrix and create sufficient separation of the teeth to form a contact when the matrix is removed. Separating rings provide strong separation and should be used with clear matrices when forming contacts because the matrices are relatively thick in comparison to most metal matrices (contrast with Fig 14 and Fig 15). (h and i) Postoperative result showing the effective management of a large bridging gap with appropriate emergence profiles. Note the evidence of the papillectomy (h) and “overmold” of the mesial cervical cavity margin on 26 (i).

A delayed wedging technique was also more likely to be used. This involves building the hip as described prior to wedging, as shown in Figures 13, 15 and 17. Wooden wedges alone can then provide sufficient separation of the teeth to form a contact when using metal matrices, as shown in Figures 14 and 15, or with the addition of a separating ring (which provides increased separation), which is especially required when using thicker clear matrices as shown in Figures 16 and 17.^3,6,29
^ Wedging also makes the matrix complex more robust. This can then allow paste composite placement under pressure without risk of destabilizing the matrix and extrusion. This contrasts with a single surface small or “claustrophobic” class II composite resin restoration, where papillectomy and delayed wedging were much less likely to be used, as a cervical seal with a wedge is often easily obtained from the start.^5^ A sectional matrix alone was much more likely to be used, and the whole cavity was more likely to be filled in one increment, presumably because there is minimal space to enable the building of a wall first as shown in Figure 15. Generally, however, building the interproximal wall first in one increment was the most commonly used approach to restore class II cavities. This allows the marginal ridge height to be set in relation to the adjacent tooth (if present), which helps to guide the accurate re-creation of occlusal anatomy and also aims to minimize finishing (see Figs 14 and 16). DSO was not often used by most experts; however, it is a useful, efficient method of building occlusal anatomy where teeth are very broken down and landmarks to guide recreation of the occlusal anatomy have been lost. This is shown in Figure 16.

**Fig 16a to p Fig16atop:**
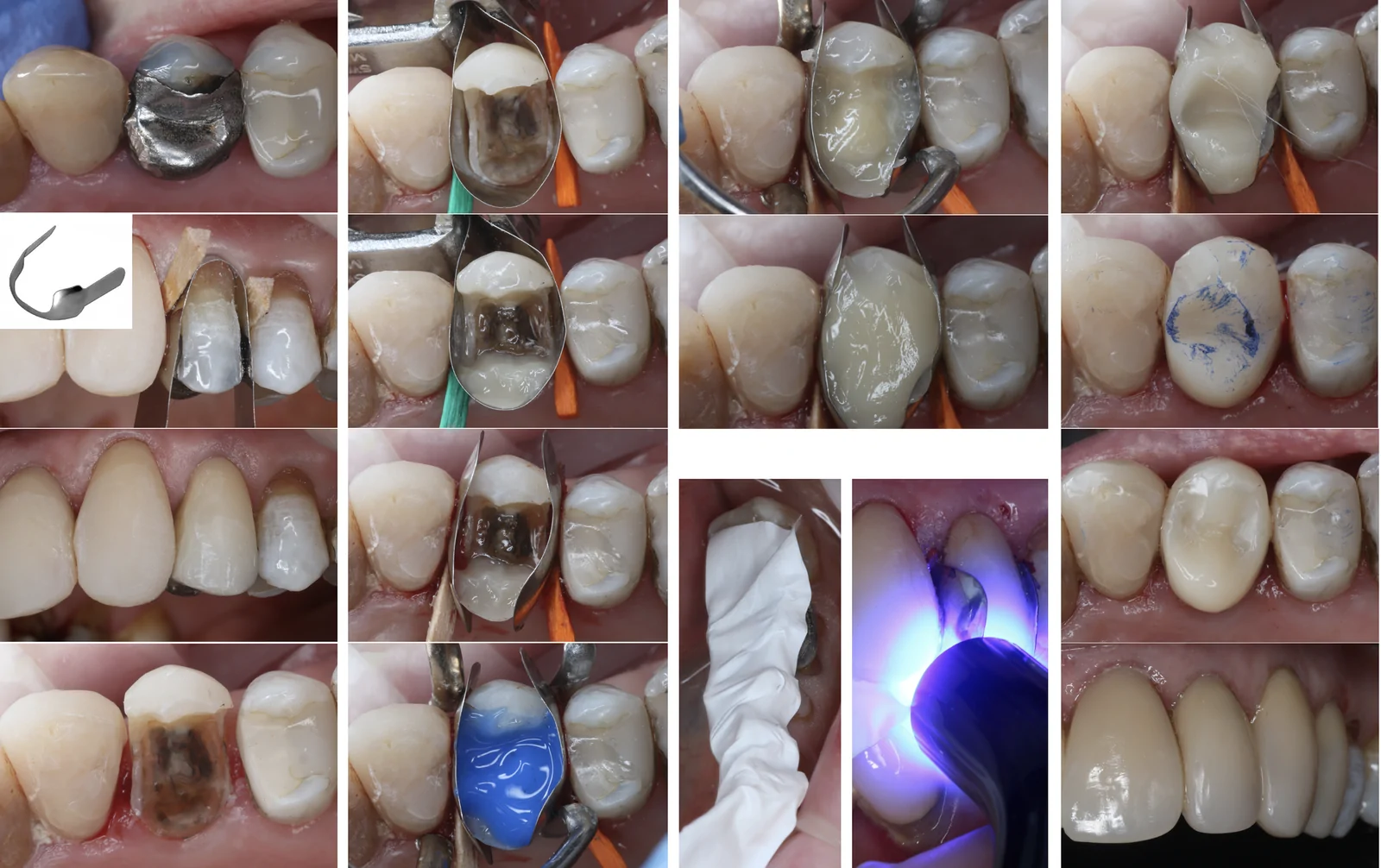
(a) Complex extended case using multiple matrices. The patient attended wishing to improve the appearance of their anterior teeth extending to the first premolars. Restoration of tooth 24 is shown. Composite restorations were chosen, and a twin anterior matrix was used (b) to provide facial coverage of 24 initially to improve aesthetics and support the thin buccal cusp (c). (d) The amalgam and caries were removed, resulting in a subgingival palatal dentin margin. An increased-curve circumferential matrix was used to seal the deep palatal margin (e) and build a palatal wall with the snow-plow application technique (f). The circumferential matrix was removed, and stiff metal sectional matrices were placed to obtain more idealized interproximal emergence profiles and contacts than if the curved circumferential matrix had been used alone (g). The patient was instructed to bite together to see if the matrices would obstruct the patient from finding their intercuspal position (ICP) (not shown) to allow the later use of the direct shaping by occlusion (DSO) technique. They did not; therefore, DSO could be used. (h) Separating rings were placed to achieve tooth separation and provide coronal stabilization of the matrices. The cavity had been contaminated, so the surfaces were particle abraded, re-etched, and bonded. (i) The marginal ridges and occlusal part of the cavity were built to slightly under full contour using a snow-plow technique. (j) The separating rings were removed and uncured composite was placed over the whole occlusal surface. PTFE tape was placed over the opposing teeth (k), and the patient was instructed to bite together (l). The unset composite was cured with the teeth in ICP. The indentation of the opposing tooth can be seen (m). The occlusion was checked (n), and the restoration was finished and polished (o and p).

Bulk-fill composite materials with reduced polymerization contraction stress can be applied in one or large increments, depending on cavity depth, and have facilitated the simplified filling of smaller “claustrophobic” cavities in one increment.^7,67
^ Allied with injection molding or snow-plow techniques, bulk-fill composites can potentially improve adaptation and reduce, rather than seal both intra-restoration and marginal voids, making this technique more predictable and efficient, especially for inexperienced operators.^7,40,51
^ Heating paste composites can also improve their flow, adaptation, and physico-mechanical properties.^23^ These techniques and materials were all variably used by experts.

Rubber dam was commonly used, but often not in more complex cases. Alternative methods of isolation were employed, including a well-adapted, curved circumferential matrix band. Though techniques exist to facilitate the placement of rubber dam in deep cases,^5,22
^ it can be difficult and there is minimal robust comparative clinical restoration survival data to support its use.^49,62
^ When placed well, it does however offer excellent isolation and other advantages, including soft tissue retraction, matrix stabilization, airway protection, limitation of bioaerosols, and improved enamel bond strengths, for example.^11,20
^ Some of these advantages are demonstrated and described in Figures 15 and 16.

Provision of cuspal coverage was variable but correlated with mesio-occluso-distal extension or more, presence of a root filling, and thin cusps.^6^ There is no consensus on this, as described in the previous paper.^6^ Guidance is provided in the following papers with multiple case examples but differing approaches to provision of direct or indirect restorations.^2,58
^


Complex direct restorative procedures are not commonly shown in textbooks; therefore, experts explore varying techniques to restore complex class II cavities with direct composite, many of which have been described or signposted here. These are potentially within the skill set of all general dental practitioners and dental therapists with training. They can help clinicians to predictably perform complex direct composite restorations.

There are a number of biases and errors which could have affected the results, and these have been described in detail elsewhere.^6^ They include the cross-sectional nature of the survey and risk of self-reporting and selection bias in the respondents whose expertise was determined by members of the core group. This paper presents the data on general use of materials and techniques simply, without further, more sophisticated statistical testing which could have been based on different groups of respondents and patterns of response, for example. Regression analyses were presented in the first paper of this series, which explored these elements in relation to the specific use of materials and techniques based on specific presented cavities and respondents’ willingness to restore these with direct composite, which was deemed more appropriate.^6^


The guidance provided is based on expert opinion and is therefore not conclusive. Expert opinion is appropriate however, due to the general paucity of long-term high-quality clinical evidence and health economic data on how cavities with different characteristics restored in different ways with different techniques perform in the long term.^4^ These can also be influenced by many factors, including the healthcare setting, the operator, and the patients who often have varying risk profiles and values.^4,15
^ Obtaining these data would be beneficial. Experts have shown good success over the long term often using some of these techniques (and alternative isolation methods to rubber dam) to perform extensive direct composite restorations, however.^30,31,52
^ They are a minimally invasive treatment option, also being less costly and time-consuming for both the patient and clinician in comparison to indirect restorations, with no robust evidence showing a clinical advantage for the extra expense. This is important given the global phase-out and decline of amalgam, which was often the material of choice for extended cavities and for those less able to pay, due to its technique insensitivity and low cost.^50^ A majority of clinicians in the UK (from a survey of > 1500) had low confidence in placing deep subgingival composites, despite 88% having received postgraduate education on posterior composites.^10^ These issues risk more teeth being extracted if primary care clinicians cannot predictably provide extended, complex direct composite restorations. Those less able to pay for more costly indirect options are more likely to be affected, potentially exacerbating already existing socioeconomic oral health inequalities.^4^


The selection of direct composite as a treatment option and its successful placement does therefore require more focused training and education at both under- and postgraduate level. This should allow clinicians to see the potential extended indications for direct composites and to upskill in a broader range of techniques to predictably approach more complex restorations.

## Conclusions

There was large variation in techniques and materials used for restoring class II cavities, with most respondents using a variety of techniques and additional equipment (eg, specialized or customized matrix bands), not commonly seen in textbooks. These varied based on the presentation. General consensus was therefore not pursued. Guidance should therefore not be too dogmatic or prescriptive. An approach of offering various attainable but predictable solutions to all clinicians is favored. However, direct composite restorations should not be contraindicated, or deemed inappropriate, in even highly complex cavities.

### Acknowledgments

Thanks to Dentsply Sirona (DS) (Dentsply DeTrey, Konstanz, Germany) for supporting the attendance of OB, TA and BL at the meeting of EFCD and ORCA, July 2023 in Egmond aan Zee (NL) and providing the contracted version of SurveyMonkey, and Alan Burgin and Kazhan Mohammed for providing the clinical case photographs in Figures 15 and 17, respectively. RF, OB, DW and TA have received lecture honoraria from DS (and other companies in the dental industry), RF has received grants from DS and TA, RF, AKL and DW are members of the DS restorative advisory board.

## References

## References

[ref1] Ahmad M, Gaikwad R, Arjumand B (2018). Comparison of two different matrix band systems in restoring two surface cavities in posterior teeth done by senior undergraduate students at Qassim University, Saudi Arabia: a randomized controlled clinical trial. Indian J Dent Res.

[ref2] Bailey O (2020). Cracked tooth syndrome management. Part 2: integrating the old with the new. Dent Update.

[ref3] Bailey O (2021). Sectional matrix solutions: the distorted truth. Br Dent J.

[ref4] Bailey O (2025). The long-term oral health consequences of an amalgam phase-out. Br Dent J.

[ref5] Bailey O, O’Connor C (2019). Papilla management in sub-gingival, interproximal, direct composite restoration: a key step to success. Br Dent J.

[ref6] Bailey O, Pfefferkorn F, Attin T, Frankenberger R, Loomans B, Lührs A (2026). Experts’ willingness to restore with direct composite: complexity, cuspal coverage, and techniques. J Adhes Dent.

[ref7] Bailey O, Shand B, Ellis I (2023). Class II composite restoration technique teaching: a randomised controlled crossover laboratory-based trial involving a novel ringless sectional matrix technique. Eur J Dent Educ.

[ref8] Bailey O, Stone S, Ternent L, Vernazza CR (2022). Public valuation of direct restorations: a discrete choice experiment. J Dent Res.

[ref9] Bailey O, Vernazza CR, Stone S, Ternent L, Roche AG, Lynch C (2022). Amalgam phase-down part 1: UK-based posterior restorative material and technique use. JDR Clin Transl Res.

[ref10] Bailey O, Vernazza CR, Stone S, Ternent L, Roche AG, Lynch C (2022). Amalgam phase-down part 2: UK-based knowledge, opinions, and confidence in the alternatives. JDR Clin Transl Res.

[ref11] Balanta-Melo J, Gutiérrez A, Sinisterra G, Díaz-Posso MdM, Gallego D, Villavicencio J (2020). Rubber dam isolation and high-volume suction reduce ultrafine dental aerosol particles: an experiment in a simulated patient. Appl Sci.

[ref12] Chana P, Orlans MC, O’Toole S, Domejean S, Movahedi S, Banerjee A (2019). Restorative intervention thresholds and treatment decisions of general dental practitioners in London. Br Dent J.

[ref13] Chuang SF, Su KC, Wang CH, Chang CH (2011). Morphological analysis of proximal contacts in class II direct restorations with 3D image reconstruction. J Dent.

[ref14] de Kuijper M, Cune MS, Özcan M, Gresnigt MMM (2023). Clinical performance of direct composite resin versus indirect restorations on endodontically treated posterior teeth: a systematic review and meta-analysis. J Prosthet Dent.

[ref15] Demarco FF, Cenci MS, Montagner AF, de Lima VP, Correa MB, Moraes RR (2023). Longevity of composite restorations is definitely not only about materials. Dent Mater.

[ref16] Duncan HF, Tomson PL, Simon S, Bjorndal L (2021). Endodontic position statements in deep caries management highlight need for clarification and consensus for patient benefit. Int Endod J.

[ref17] Edwards D, Bailey O, Stone S, Duncan H (2021). The management of deep caries in UK primary care: a nationwide questionnaire-based study. Int Endod J.

[ref18] Edwards D, Bailey O, Stone SJ, Duncan H (2021). How is carious pulp exposure and symptomatic irreversible pulpitis managed in UK primary dental care. Int Endod J.

[ref19] El-Shamy H, Sonbul H, Alturkestani N, Tashkandi A, Loomans BA, Dörfer C (2019). Proximal contact tightness of class II bulk-fill composite resin restorations: an in vitro study. Dent Mater J.

[ref20] Falacho RI, Melo EA, Marques JA, Ramos JC, Guerra F, Blatz MB (2023). Clinical evaluation of the effect of rubber dam isolation on bond strength to enamel. J Esthet Restor Dent.

[ref21] Ferracane JL, Lawson NC (2021). Probing the hierarchy of evidence to identify the best strategy for placing class II dental composite restorations using current materials. J Esthet Restor Dent.

[ref22] Fichera G, Mazzitelli C, Picciariello V, Maravic T, Josic U, Mazzoni A (2024). Structurally compromised teeth. Part II: a novel approach to peripheral build up procedures. J Esthet Restor Dent.

[ref23] Freedman G, Krejci I (2004). Warming up to composites. Compend Contin Educ Dent.

[ref24] Frese C, Wolff D, Staehle HJ (2014). Proximal box elevation with resin composite and the dogma of biological width: clinical R2-technique and critical review. Oper Dent.

[ref25] Gomes IA, Filho EMM, Mariz DCBR, Borges AH, Tonetto MR, Firoozmand LM (2015). In vivo evaluation of proximal resin composite restorations performed using three different matrix systems. J Contemp Dent Pract.

[ref26] Hahn B, Haubitz I, Krug R, Krastl G, Soliman S (2022). Influence of matrix type on marginal gap formation of deep class II bulk-fill composite restorations. Int J Environ Res Public Health.

[ref28] Heintze SD, Rousson V (2012). Clinical effectiveness of direct class II restorations – a meta-analysis. J Adhes Dent.

[ref29] Hellie CM, Charbeneau GT, Craig RG, Brandau HE (1985). Quantitative evaluation of proximal tooth movement effected by wedging – a pilot study. J Prosthet Dent.

[ref30] Hofsteenge JW, Fennis WMM, Kuijs RH, Özcan M, Cune MS, Gresnigt MMM (2023). Clinical survival and performance of premolars restored with direct or indirect cusp-replacing resin composite restorations with a mean follow-up of 14 years. Dent Mater.

[ref31] Hofsteenge JW, Scholtanus JD, Ozcan M, Nolte IM, Cune MS, Gresnigt MMM (2023). Clinical longevity of extensive direct resin composite restorations after amalgam replacement with a mean follow-up of 15 years. J Dent.

[ref32] Josic U, D’Alessandro C, Miletic V, Maravic T, Mazzitelli C, Jacimovic J (2023). Clinical longevity of direct and indirect posterior resin composite restorations: an updated systematic review and meta-analysis. Dent Mater.

[ref34] Kampouropoulos D, Paximada C, Loukidis M, Kakaboura A (2010). The influence of matrix type on the proximal contact in class II resin composite restorations. Oper Dent.

[ref35] Kopperud SE, Tveit AB, Gaarden T, Sandvik L, Espelid I (2012). Longevity of posterior dental restorations and reasons for failure. Eur J Oral Sci.

[ref36] Laske M, Opdam NJM, Bronkhorst EM, Braspenning JCC, Huysmans M (2016). Ten-year survival of class II restorations placed by general practitioners. JDR Clin Transl Res.

[ref37] Laske M, Opdam NJM, Bronkhorst EM, Braspenning JCC, Huysmans MCDNJM (2019). Risk factors for dental restoration survival: a practice-based study. J Dent Res.

[ref38] Laske M, Opdam NJM, Bronkhorst EM, Braspenning JCC, van der Sanden WJM, Huysmans MCDNJM (2019). Minimally invasive intervention for primary caries lesions: are dentists implementing this concept. Caries Res.

[ref39] Lehmann A, Nijakowski K, Jankowski J (2025). Clinical difficulties related to direct composite restorations: a multinational survey. Int Dent J.

[ref40] Leinonen KM, Leinonen J, Bolstad NL, Tanner T, Al-Haroni M, Johnsen JAK (2023). Procedure time and filling quality for bulk-fill base and conventional incremental composite techniques – a randomised controlled in vitro trial. J Dent.

[ref41] Loomans B, Hilton T (2016). Extended resin composite restorations: techniques and procedures. Oper Dent.

[ref42] Loomans B, Mendes FM, Vinayahalingam S, Xi T, Opdam N, Kreulen CM (2024). Challenges in conducting clinical research in primary care dentistry. J Dent.

[ref43] Loomans BAC, Opdam NJM, Bronkhorst EM, Roeters FJM, Dörfer CE (2007). A clinical study on interdental separation techniques. Oper Dent.

[ref44] Loomans BAC, Opdam NJM, Roeters FJM, Bronkhorst EM, Huysmans MCDNJM (2009). Restoration techniques and marginal overhang in class II composite resin restorations. J Dent.

[ref45] Loomans BAC, Roeters FJM, Opdam NJM, Kuijs RH (2008). The effect of proximal contour on marginal ridge fracture of class II composite resin restorations. J Dent.

[ref46] Lucarotti PSK, Burke FJT (2018). The ultimate guide to restoration longevity in England and Wales. Part 1: methodology. Br Dent J.

[ref47] Lührs A, Jacker-Guhr S, Herrmann P (2018). Think outside the box! Proximal box elevation for managing deep proximal lesions. DZZ Int.

[ref48] Lynch CD, Farnell DJJ, Stanton H, Chestnutt IG, Brunton PA, Wilson NHF (2018). No more amalgams: use of amalgam and amalgam alternative materials in primary dental care. Br Dent J.

[ref49] Miao C, Yang X, Wong MCM, Zou J, Zhou X, Li C (2021). Rubber dam isolation for restorative treatment in dental patients. Cochrane Database Syst Rev.

[ref52] Opdam NJM, Bronkhorst EM, Loomans BAC, Huysmans MCDNJM (2010). 12-year survival of composite vs. amalgam restorations. J Dent Res.

[ref53] Opdam NJM, Collares K, Hickel R, Bayne SC, Loomans BA, Cenci MS (2018). Clinical studies in restorative dentistry: new directions and new demands. Dent Mater.

[ref54] Opdam NJM, Roeters JJM, de Boer T, Pesschier D, Bronkhorst E (2003). Voids and porosities in class I micropreparations filled with various resin composites. Oper Dent.

[ref55] Opdam NJM, Roeters JJM, Kuijs R, Burgersdijk RCW (1998). Necessity of bevels for box only class II composite restorations. J Prosthet Dent.

[ref56] Opdam NJM, Skupien JA, Kreulen CM, Roeters JJM, Loomans BAC, Huysmans MCDNJM (2016). Case report: a predictable technique to establish occlusal contact in extensive direct composite resin restorations: the DSO-technique. Oper Dent.

[ref57] Pandya JK, Wheatley J, Bailey O, Taylor G, Geddis-Regan A, Edwards D (2024). Exploring deep caries management and barriers to the use of vital pulp treatments by primary care dental practitioners. Int Endod J.

[ref58] Peumans M, Politano G, Van Meerbeek B (2020). Effective protocol for daily high-quality direct posterior composite restorations. Cavity preparation and design. J Adhes Dent.

[ref59] Peumans M, Van Meerbeek B, Asscherickx K, Simon S, Abe Y, Lambrechts P (2001). Do condensable composites help to achieve better proximal contacts. Dent Mater.

[ref60] Peumans M, Venuti P, Politano G, Van Meerbeek B (2021). Effective protocol for daily high-quality direct posterior composite restorations. The interdental anatomy of the class-2 composite restoration. J Adhes Dent.

[ref61] Raedel M, Hartmann A, Bohm S, Priess HW, Samietz S, Konstantinidis I (2017). Four-year outcomes of restored posterior tooth surfaces – a massive data analysis. Clin Oral Investig.

[ref62] Raskin A, Setcos JC, Vreven J, Wilson NH (2000). Influence of the isolation method on the 10-year clinical behaviour of posterior resin composite restorations. Clin Oral Investig.

[ref63] Saber MH, Loomans BAC, El Zohairy A, Dörfer CE, El-Badrawy W (2010). Evaluation of proximal contact tightness of class II resin composite restorations. Oper Dent.

[ref64] Schwendicke F, Foster Page LA, Smith LA, Fontana M, Thomson WM, Baker SR (2018). To fill or not to fill: a qualitative cross-country study on dentists’ decisions in managing non-cavitated proximal caries lesions. Implement Sci.

[ref65] Schwendicke F, Opdam N (2018). Clinical studies in restorative dentistry: design, conduct, analysis. Dent Mater.

[ref67] Van Ende A, De Munck J, Lise DP, Van Meerbeek B (2017). Bulk-fill composites: a review of the current literature. J Adhes Dent.

[ref68] Vidnes-Kopperud S, Tveit AB, Gaarden T, Sandvik L, Espelid I (2009). Factors influencing dentists’ choice of amalgam and tooth-colored restorative materials for class II preparations in younger patients. Acta Odontol Scand.

[ref69] Wirsching E, Loomans BAC, Klaiber B, Dörfer CE (2011). Influence of matrix systems on proximal contact tightness of 2- and 3-surface posterior composite restorations. J Dent.

[ref70] Wolff D, Frese C, Frankenberger R, Haak R, Braun A, Krämer N (2024). Direct composite restorations on permanent teeth in the anterior and posterior region – an evidence-based clinical practice guideline – part 1: indications for composite restorations. J Adhes Dent.

